# Mesenchymal Stromal Cells Increase the Natural Killer Resistance of Circulating Tumor Cells via Intercellular Signaling of cGAS‐STING‐IFNβ‐HLA

**DOI:** 10.1002/advs.202400888

**Published:** 2024-04-18

**Authors:** Ye Yi, Guihui Qin, Hongmei Yang, Hao Jia, Qibing Zeng, Dejin Zheng, Sen Ye, Zhiming Zhang, Tzu‐Ming Liu, Kathy Qian Luo, Chu‐Xia Deng, Ren‐He Xu

**Affiliations:** ^1^ Center of Reproduction, Development and Aging Cancer Center and Institute of Translational Medicine Faculty of Health Sciences University of Macau Taipa Macao SAR 999078 China; ^2^ Ministry of Education Frontiers Science Center for Precision Oncology University of Macau Taipa Macao SAR 999078 China

**Keywords:** cGAS‐STING‐IFNβ‐HLA pathway, circulating tumor cells, mesenchymal stromal cells, natural killer cells

## Abstract

Circulating tumor cells (CTCs) shed from primary tumors must overcome the cytotoxicity of immune cells, particularly natural killer (NK) cells, to cause metastasis. The tumor microenvironment (TME) protects tumor cells from the cytotoxicity of immune cells, which is partially executed by cancer‐associated mesenchymal stromal cells (MSCs). However, the mechanisms by which MSCs influence the NK resistance of CTCs remain poorly understood. This study demonstrates that MSCs enhance the NK resistance of cancer cells in a gap junction‐dependent manner, thereby promoting the survival and metastatic seeding of CTCs in immunocompromised mice. Tumor cells crosstalk with MSCs through an intercellular cGAS‐cGAMP‐STING signaling loop, leading to increased production of interferon‐β (IFNβ) by MSCs. IFNβ reversely enhances the type I IFN (IFN‐I) signaling in tumor cells and hence the expression of human leukocyte antigen class I (HLA‐I) on the cell surface, protecting the tumor cells from NK cytotoxicity. Disruption of this loop reverses NK sensitivity in tumor cells and decreases tumor metastasis. Moreover, there are positive correlations between IFN‐I signaling, HLA‐I expression, and NK tolerance in human tumor samples. Thus, the NK‐resistant signaling loop between tumor cells and MSCs may serve as a novel therapeutic target.

## Graphical abstract

This study shows that circulating tumor cells, before dissemination from a primary tumor, are empowered to escape NK cell surveillance and form metastasis by cross‐talking with mesenchymal stromal cells in the tumor microenvironment via an intercellular cGAS‐cGAMP‐STING‐IFNβ‐HLA‐I signaling loop. The new mechanism may provide novel therapeutic targets.

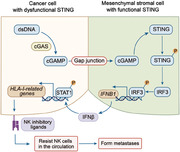

## 1 Introduction

Circulating tumor cells (CTCs) that disseminate from a primary tumor to distant organs through the bloodstream are the major source of metastatic tumors and cause 90% of cancer‐associated deaths.^[^ ^1^, ^2^ ^]^ However, the majority of CTCs perish due to hemodynamic shear stress, anoikis, and immunosurveillance^[^ ^3^, ^4^, ^5^ ^]^ and only a small proportion (<0.02%) of CTCs can overcome these obstacles and function as disseminated seeds to initiate tumor metastasis.^[^ ^6^, ^7^ ^]^ Thus, it is crucial to understand the mechanisms by which CTCs survive and initiate metastasis. Identifying potential interventions to disrupt these mechanisms holds great importance.

The immune system plays a pivotal role in all stages of tumor progression.^[^ ^8^ ^]^ As CTCs appear transiently in the circulation, effective immunosurveillance of transformed cells requires quick recognition and direct cytotoxicity by immune cells.^[^ ^9^ ^]^ Natural killer (NK) cells, which were discovered in the 1970s, are a type of lymphoid cells belonging to the innate immune system. NK cells have the ability to quickly eliminate malignant or virally infected cells without the need for prior sensitization or priming.^[^ ^10^ ^]^ Therefore, NK cells exert a predominant role in immunosurveillance against CTCs compared to that of other cytotoxic immune cells.^[^ ^9^, ^11^, ^12^ ^]^ The functions of NK cells are regulated by an array of activating and inhibitory receptors that interact with specific ligands on target cells.^[^ ^13^ ^]^ When inhibitory signals override activating signals, the NK cell cannot be activated.^[^ ^13^ ^]^ Thus, CTCs can evade NK cytotoxicity by upregulating the expression of the inhibitory ligands or reducing the expression of the activating ligands.^[^ ^9^, ^14^, ^15^ ^]^

Although the characteristics of tumor cells are influenced mainly by various cell‐intrinsic mechanisms, both the tumor microenvironment (TME) and systemic processes also play crucial roles.^[^ ^16^ ^]^ The TME consists of blood vessels, immune cells, fibroblasts, signaling molecules, and the extracellular matrix.^[^ ^17^ ^]^ Mesenchymal stromal cells (MSCs) are ubiquitous in vascularized organs and tissues. MSCs are also a major component of the TME in both primary and metastatic tumors and play a key role in promoting tumor progression.^[^ ^18^, ^19^ ^]^ The spatially close relationship between MSCs and tumor cells allows them to constantly interact via cell‐cell contact and secreted factors.^[^ ^20^ ^]^ MSCs in primary tumor sites drive tumor cells toward an invasive and prometastatic state.^[^ ^21^ ^]^ However, it remains unclear whether MSCs confer NK resistance to cancer cells within the TME, potentially facilitating the evasion of CTCs from NK cell‐mediated immunosurveillance in the circulation.

We hypothesize here that MSCs contribute to the development of NK resistance in CTCs before intravasation, thereby promoting metastasis formation. To test this hypothesis, we cocultured human breast cancer organoids with MSCs. We found that MSCs increased cancer cell resistance to NK cells in a gap junction‐dependent manner, thereby enhancing their survival in vitro and in vivo. Furthermore, we demonstrated that MSC‐induced NK resistance in cancer cells was related to an intercellular loop of cGAS‐STING‐IFNβ‐HLA signaling, consistent with the findings of clinical tumor sample analysis. Importantly, intervention in this loop reduced NK resistance and impeded tumor metastasis.

## 2 Results

### 2.1 MSCs Interact with Breast Cancer Cells from Patients and Increase Cancer Cell Resistance to NK Cytotoxicity

**Figure 1:**
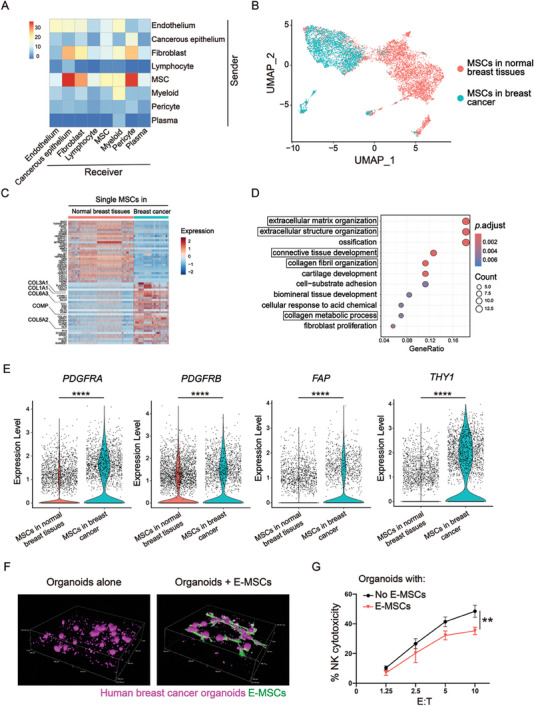
MSCs interact with cancer cells in human primary breast cancer and increase the natural killer (NK) resistance of the cancer cells. A) CellChat analysis showing the mutual interactions among the main cellular components in human breast cancer. B) UMAP visualization of 5, 047 MSCs from the human normal breast tissues and 2, 675 MSCs from breast cancer tissues. C) Heatmap showing the top 30 upregulated and top 30 downregulated genes between MSCs from normal breast tissues and those from breast cancer tissues. The names of genes related to collagen activation are enlarged. D) GO analysis of the two groups of MSCs with framed terms related to collagen activation or matrix metalloproteinases. E) Violin plots showing the expression of protumorigenic genes *PDGFRA*, *PDGFRB*, *FAP* and *THY1* in the two groups of MSCs. The Mann‒Whitney two‐sided test was used to test the significance of differences in gene expression level between the two groups. ^****^ *p* < 0.0001. F) 3D images of human breast cancer organoids cultured alone (left) or cultured with E‐MSCs (right). G) NK assay of tumor cells collected from F. The data are presented as the means ± standard deviations (SD). N = 3, ^**^ *p* < 0.01 for two‐way ANOVA with multiple comparisons.

It is known that complicated interactions among TME components play a critical role in tumor progression.^[^ ^22^ ^]^ Pal, et al. reported a single‐cell RNA‐sequencing (scRNA) dataset on 69 distinct surgical tissue specimens from 55 patients with breast cancer versus normal breast tissues from 18 women with reduction mammoplasties, and profiled dynamic transcriptomic changes in breast cancer across different stages of tumor progression.^[^ ^23^ ^]^ To elucidate the novel pro‐tumorigenic mechanisms underlying the interactions, we utilized this dataset to dissect the cellular interactions among TME components and cancer cells. We used canonical markers^[^ ^24^, ^25^ ^]^ to first classify epithelial cells, fibroblasts, lymphocytes, myeloid cells, endothelial cells, and MSCs in both groups of samples (Figure S1A, Supporting Information). Breast cancer mainly arises from epithelial cells and is primarily driven or characterized by high copy number variations (CNVs) in the genomic DNA.^[^ ^26^ ^]^ Thus, we estimated single‐cell CNV profiles using inferCNV^[^ ^27^ ^]^ to distinguish CNV‐high epithelial cells, indicative of cancerous epithelial cells, from CNV‐low normal epithelial cells (Figure S1B, Supporting Information). Then, we employed CellChat analysis^[^ ^28^ ^]^ to assess interactions among different cellular components in the TME. The results revealed that cancerous epithelial cells actively interacted with all the other cellular components, but MSCs presented the most prolific interactions with the other cellular components, including cancerous epithelial cells (**Figure** **1**A). These data indicate that MSCs are active components that interact with cancer cells in the TME.

Next, we compared the transcriptomes of MSCs from the breast cancer and normal breast tissues (Figure 1B). Over 2000 differentially expressed genes (DEGs) were identified between MSCs from the two sources. The upregulated DEGs in breast cancer MSCs included genes related to collagen activation and matrix metalloproteinase (Figure 1C,D), which is indicative of MSC activation in the TME,^[^ ^24^ ^]^ and genes involved in angiogenesis and immunomodulation, e.g., *PDGFRA*, *PDGFRB*, *FAP*, and *THY1*, which are critical for tumor progression.^[^ ^29^, ^30^, ^31^ ^]^ These data suggest that MSCs in the TME undergo activation to support tumorigenesis.

It has been widely reported that MSCs in the TME promote tumor metastasis^[^ ^21^, ^32^ ^]^ and that NK cells play a pivotal role in controlling metastasis by eliminating CTCs.^[^ ^9^, ^14^ ^]^ To test whether MSCs can modify the response of CTCs to NK cytotoxicity before departure from a primary tumor, we cocultured organoids formed by human primary breast cancer cells^[^ ^33^ ^]^ with MSCs derived from the ENVY human embryonic stem cell (hESC) line^[^ ^34^ ^]^ in a 3D system. To distinguish the two cell sources, we stained the breast cancer cells with TO‐PRO‐3 (red) in contrast to hESC‐derived MSCs (E‐MSCs), which expresse green fluorescent protein (GFP)^[^ ^35^ ^]^ (Figure 1F). In the presence of E‐MSCs, the cancer cells tended to aggregate in the center of the organoids, while the E‐MSCs lined along the margins (Figure 1F). After 3 days of coculture, the organoids were dissociated, and the cancer cells were isolated by sorting. Then, the sorted cancer cells were mixed with human primary NK cells to assess cell lysis through the Calcein‐AM assay of the supernatants.^[^ ^36^ ^]^ Following coculture with E‐MSCs, the cancer cells exhibited reduced lysis by NK cells compared to that of cancer cells without coculture (Figure 1G). These data suggest that MSCs confer increased NK resistance to cancer cells in the organoids.

### 2.2 The Ability of MSCs to Increase NK Resistance in Human Cancer Cells Depends on Cell‒Cell Contact and Gap Junctions

**Figure 2:**
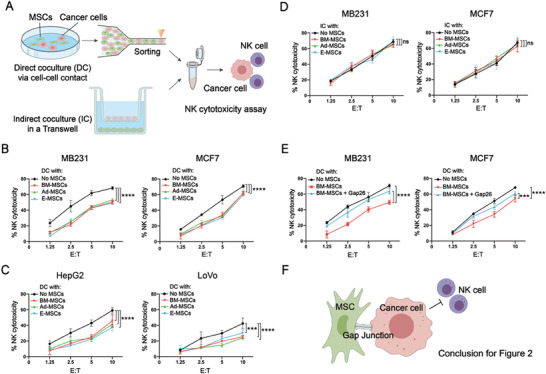
MSC‐DC‐mediated increase in NK resistance in human cancer cells relies on cell‒cell contact and gap junctions. A) Schematic representation of cancer cells in DC or IC with MSCs and subsequent NK assay with human primary NK cells. B–D) NK assay of human cancer cells following DC B and C) and IC D) treatment with vehicle or MSCs from various sources. E) NK assay of human cancer cells following DC treatment with BM‐MSCs and Gap26. F) Schematic summary of MSC‐enhanced NK resistance of cancer cells during DC. The data are presented as the means ± standard deviations (SD). N = 3, ^***^ *p* < 0.001 and ^****^ *p* < 0.0001 per two‐way ANOVA with multiple comparisons.

To elucidate the mechanism underlying MSC‐induced NK resistance in cancer cells, we conducted direct coculture (DC) and indirect coculture (IC) of human cancer cell lines and MSCs in vitro to mimic crosstalk between both cell types within the TME. Subsequently, the cancer cells sorted from the DC or collected from the IC were mixed with NK cells to assess cell lysis (**Figure** **2**A). These experiments employed two human breast cancer cell lines, MDA‐MB‐231 (MB231) and MCF7, along with MSCs derived from three sources: human bone marrow (BM), adipose tissue (Ad), and human embryonic stem cells (hESCs). As shown in Figure 2B, MSC‐DC with either breast cancer cell line reduced the lysis of the cancer cells by the NK cells compared to that of MSCs without DC. Similar results were observed for the human liver cancer cell line HepG2 and the colon cancer cell line LoVo following MSC‐DC (Figure 2C).

As MSCs also influence tumor progression via secreted cytokines, exosomes, extracellular vesicles, and other factors,^[^ ^37^ ^]^ we further tested the IC of MB231 and MCF7 cancer cells with MSCs in Transwell plates (Figure 2A). Unlike MSC‐DC, MSC‐IC failed to confer any protective effects against NK cytotoxicity to cancer cells (Figure 2D). These results suggest that coculture with MSCs increases cancer cell resistance to NK cells in a manner dependent on direct cell‐cell contact.

Among cell‒cell adhesion molecules, gap junctions composed of the connexin family members^[^ ^38^ ^]^ serve as central channels facilitating the transmission of ions, essential metabolites, and secondary messengers such as Ca^2+^, cAMP, and cGAMP.^[^ ^39^ ^]^ Gap junctions can establish connections between different cell types.^[^ ^40^ ^]^ Hence, we tested whether gap junctions mediate molecule exchange between MSCs and cancer cells during DC to increase NK resistance in cancer cells. Connexin 43 is a member of the connexin family that is highly expressed in BM‐MSCs.^[^ ^41^, ^42^ ^]^ The gap junctions formed by connexin 43 can be inhibited by the mimetic peptide, Gap26.^[^ ^43^ ^]^ Therefore, we added 0.25 mg ml^−1^ of Gap26 to the DC of BM‐MSCs and breast cancer cell lines and observed that Gap26 markedly reversed the NK resistance of the cancer cells following MSC‐DC (Figure 2E). Overall, these data indicate that the ability of MSCs to increase the NK resistance of cancer cells primarily depends on molecular transfer via gap junctions between the two cell types (Figure 2F).

### 2.3 MSCs Increase CTC Resistance to NK Cells and Metastasis In Vivo

**Figure 3:**
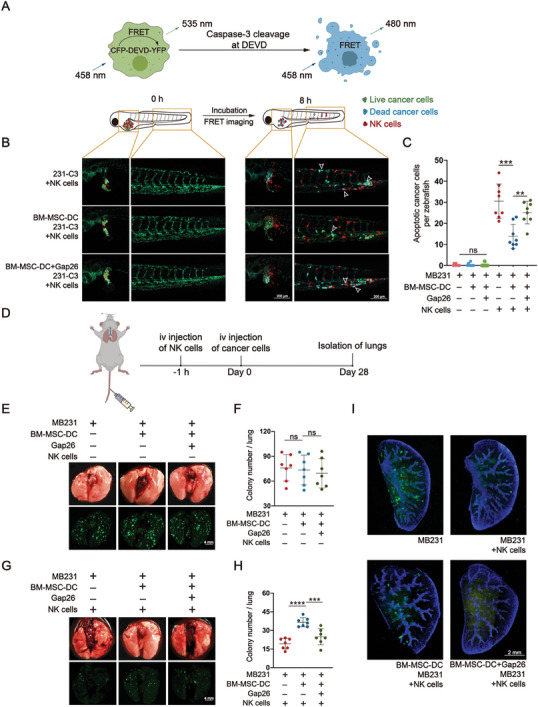
MSC‐DC increases the NK resistance of human cancer cells in zebrafish and mice. A) Schematic for FRED‐based visualization of human CTCs expressing an apoptotic sensor for NK cell killing in zebrafish. B) Representative microscopy images of zebrafish at 0 and 8 h after injection of 231‐C3 cells and NK‐92MI‐tdTomato cells, following DC with or without BM‐MSCs before the injection. The 231‐C3 cells were in green when alive and turned blue upon apoptosis, and the NK cells were in red. White arrowheads denote blue/apoptotic cells. The vasculature of the zebrafish is also shown in green. Scale bar: 200 µm. C) Quantification of apoptotic 231‐C3 cells in B. N = 8, ^**^ *p* < 0.01 and ^***^ *p* < 0.001 per two‐tailed unpaired *t* test. D) Schematic representation of nodule formation of human CTCs in mouse lungs. E and G) Representative bright‐field (upper) and fluorescent (bottom) images of nodule formation in the lungs of NOD/SCID mice at 28 days following i.v. injection of GFP^+^ MB231 following DC with or without BM‐MSCs and Gap26. Vehicle E) or human primary NK cells G) were injected i.v. 1 h before the cancer cell injection. Scale bar: 4 mm. F and H) Quantification of the lung nodules per mouse in E and G, respectively. N = 7, ^***^ *p* < 0.001 and ^****^ *p* < 0.0001 per two‐tailed unpaired *t* test. I) Representative two‐photon microscopy images of GFP^+^ MB231 cells in the cleared lungs of mice in E and G. Scale bar: 2 mm.

To mimic the confrontation of CTCs and NK cells in the circulation and to examine the effect of MSCs on the resistance of CTCs to NK cells in vivo, we established a NK assay in zebrafish, in which human cancer cells and NK cells were injected and allowed to circulate for 8 h. We genetically introduced a caspase‐3 responsive biosensor, C3, into MB231 (named 231‐C3), which can then change from green to blue upon apoptosis, based on fluorescence resonance energy transfer (FRET).^[^ ^44^ ^]^ Thus, we could directly detect the real‐time apoptosis of the injected cells to mimic the death of CTCs in the circulation (**Figure** **3**A). For better visualization of the interaction between cancer cells and NK cells in the circulation and subsequent apoptosis of cancer cells, we utilized zebrafish with endothelial cells expressing EGFP, rendering their blood vessels visible in green.^[^ ^45^ ^]^ After DC with BM‐MSCs for 3 days, approximately one hundred 231‐C3 cells were collected and injected into the common cardinal vein of the zebrafish. At 8 h postinjection, 231‐C3 cells were found to circulate in the host with few blue cells (indicating apoptotic cells), regardless of the presence or absence of MSC‐DC (Figure 3B,C; Figure S2, Supporting Information). These findings suggest that MSC‐DC does not affect the viability of CTCs in the circulation.

Next, we coinjected 231‐C3 cells with NK‐92MI cells expressing the tandem dimer Tomato (tdTomato).^[^ ^46^ ^]^ Notably, all groups showed blue/apoptotic 231‐C3 cells; however, fewer were observed in zebrafish injected with 231‐C3 cells sorted from MSC‐DC than in those without MSC‐DC (Figure 3B,C). The introduction of Gap26 partially reversed this effect (Figure 3B,C). These observations suggest that MSC‐DC also increases the NK resistance of cancer cells in vivo via communication between MSCs and cancer cells via gap junctions.

To investigate whether increased NK resistance confers an advantage to CTCs during metastasis formation, we conducted a tail vein metastasis assay in non‐obese diabetic/severe combined immunodeficient (NOD/SCID) mice.^[^ ^47^ ^]^ Specifically, GFP^+^ MB231 cells, sorted from the DC with human BM‐MSCs in the presence or absence of the gap junction inhibitor Gap26 for 3 days, were intravenously (i.v.) injected into the mice. Subsequently, GFP^+^ metastatic nodules on the lung surface were photographed and counted on Day 28 postinjection (Figure 3D). Similar numbers of lung nodules were observed in the injected mice in all the groups (Figure 3E,F), suggesting that MSC‐DC treatment and gap junction blockade did not influence CTC survival or metastasis in the lungs.

Next, human primary NK cells were injected 1 h prior to the i.v. injection of cancer cells. As expected, NK cells killed most of the CTCs, thus dramatically reducing the nodule formation in the mouse lungs (Figure 3G,H). However, more lung nodules (1.89‐fold) were observed in the group of MB231 with MSC‐DC than in the group without MSC‐DC, and this difference was reduced by Gap26 (Figure 3G,H). Furthermore, we cleared the lungs to visualize all the GFP^+^ nodules in the transparent lungs. Consistently, more GFP^+^ nodules were observed in the group of MB231 with MSC‐DC than in the control group (Figure 3I). Together, these results suggest that MSC‐DC, which are dependent on gap junctions, increases the NK resistance and lung metastasis of CTCs.

### 2.4 MSCs Increase IFN‐I Signaling and HLA‐I Expression in Cancer Cells

**Figure 4:**
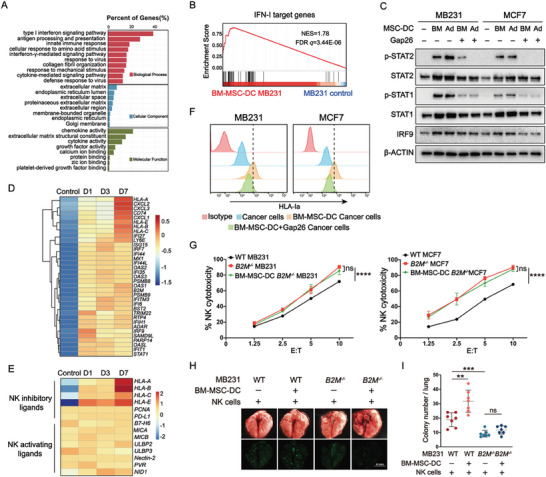
Elevated IFN‐I response and HLA‐I expression in cancer cells following DC with MSCs. A) GO analysis of RNA‐seq data for MB231 cancer cells following DC with or without BM‐MSCs for 3 days. The red, blue, and green bars represent enriched terms for biological processes, cellular components, and molecular functions, respectively. B) GSEA results showing the enrichment of IFN‐I target genes in MB231 cells following DC with or without BM‐MSCs for 3 days. C) Western blotting showing STAT1 and STAT2 phosphorylation and IRF9 expression in MB231 and MCF7 cells following DC for 3 days with or without BM‐MSCs and Ad‐MSCs or Gap26. D and E) GO analysis‐derived heatmaps displaying genes related to response D) and encoding NK‐inhibitory and ‐activating ligands E) in MB231 cells following DC with BM‐MSCs for 1, 3, and 7 days. F) Flow cytometry for expression of HLA‐Ia molecules on the surface of MB231 and MCF7 cells following DC with BM‐MSCs and Gap26 for 3 days. G) NK assay on WT and *B2M^−/−^* cancer cells following DC with BM‐MSCs for 3 days. N = 3. Two‐way ANOVA with multiple comparisons was performed. H) Representative bright‐field (upper) and fluorescent (bottom) images of nodule formation in the lungs of NOD/SCID mice at 28 days post i.v. injection with GFP^+^ WT or *B2M^−/−^* MB231 cells following DC with or without BM‐MSCs. Human primary NK cells were i.v. injected 1 h before the cancer cell injection. Scale bar: 4 mm. I) Quantification of the lung nodules per mouse in H. N = 7. ^**^ *p* < 0.01 per two‐tailed unpaired *t* test. Data are presented as means ± SD.

To determine the factors that contributed to the increased resistance to NK cell killing, we collected MB231 cells from the DC with BM‐MSCs for 3 days and analyzed the gene expression profile profiles via RNA‐seq. Gene Ontology (GO) enrichment analysis revealed significant enrichments of genes related to the IFN‐I response, antigen processing and antigen presentation in MB231 cells with prior MSC‐DC (**Figure** **4**A). Moreover, Gene Set Enrichment Analysis (GSEA) revealed a marked upregulation of IFN‐I target genes in MB231 cells following DC with MSCs (Figure 4B and Table S3, Supporting Information). We confirmed the activation of IFN‐I signaling in cancer cells with MSC‐DC, as reflected by increased phosphorylation of STAT1 and STAT2^[^ ^48^ ^]^ and increased IRF9 expression,^[^ ^48^ ^]^ which was reduced by Gap26 (Figure 4C). Other IFN‐I related genes were also increased in MB231 cells at various time points following MSC‐DC (Figure 4D). These data suggest that gap junction‐mediated intercellular communication between MSCs and cancer cells triggers the activation of IFN‐I signaling in cancer cells.

It is known that a weak and chronic IFN‐I signaling modulates the expression of immune checkpoint molecules, such as HLA molecules, in cancer cells, thereby altering their susceptibility to immune cells.^[^ ^49^ ^]^ Classical HLA‐I (HLA‐Ia) molecules, including HLA‐A, HLA‐B, and HLA‐C, bind to killer cell immunoglobulin‐like receptors (KIRs) on NK cells, providing inhibitory signals for NK cell functions.^[^ ^50^ ^]^ The non‐classical HLA‐I (HLA‐Ib) molecule, HLA‐E, interacts with CD94/NKG2 on NK cells and provides inhibitory signals to prevent NK cell activation.^[^ ^51^, ^52^ ^]^ Following MSC‐DC, there was a time‐dependent increase in the expression of these NK inhibitory ligands, HLAs, in MB231 cells, whereas the expression of other NK inhibitory ligands such as PCNA^[^ ^53^, ^54^ ^]^ and PD‐L1,^[^ ^55^, ^56^ ^]^ along with NK‐activating ligands including the MICA/B^[^ ^57^ ^]^ and ULBP family,^[^ ^57^, ^58^ ^]^ remained at basal levels. These results were confirmed at both the mRNA (Figure 4E) and protein levels (Figures 4F; Figure S3A–C, Supporting Information), suggesting that elevated IFN‐I signaling and HLA‐I expression in cancer cells, following MSC‐DC treatment, are associated with the increased NK resistance in cancer cells.

To elucidate the role of HLA‐I molecules in increased NK resistance in cancer cells, we knocked out *β_2_ microglobulin* (*B2M*) in MB231 and MCF7 cells using the CRISPR‐Cas9 technology (Figure S3D, Supporting Information). B2M is a shared component of all HLA‐I molecules and is crucial for HLA‐I assembly on the cell surface.^[^ ^59^ ^]^ Therefore, the *B2M^−/−^* cancer cells lack HLA‐I molecules on their cell surface (Figure S3E, Supporting Information). Due to the absence of these inhibitory ligands, the *B2M^−/−^* cancer cells exhibited increased susceptibility to NK cells compared with that of the WT control (Figure 4G). More importantly, we found that the DC with MSCs did not change the NK resistance of *B2M^−/−^* cancer cells (Figure 4G; Figure S3F, Supporting Information). This finding implies that MSC‐DC‐induced NK resistance is dependent on the increased levels of the HLA‐I molecules on the cancer cell surface. Next, we i.v. injected WT or *B2M^−/−^* MB231 cells i.v. into NOD/SCID mice, followed by injection of human primary NK cells 1 h later. MSC‐DC only increased the number of metastatic nodules in mice injected with the WT, but not in those injected with *B2M^−/−^* MB231 cells (Figure 3H,I). Together, the above data suggest that elevated levels of the HLA‐I molecules on CTCs are responsible for increased NK resistance and metastasis.

### 2.5 MSC‐Secreted IFNβ Increases HLA‐I Expression and NK Resistance of Cancer Cells

**Figure 5:**
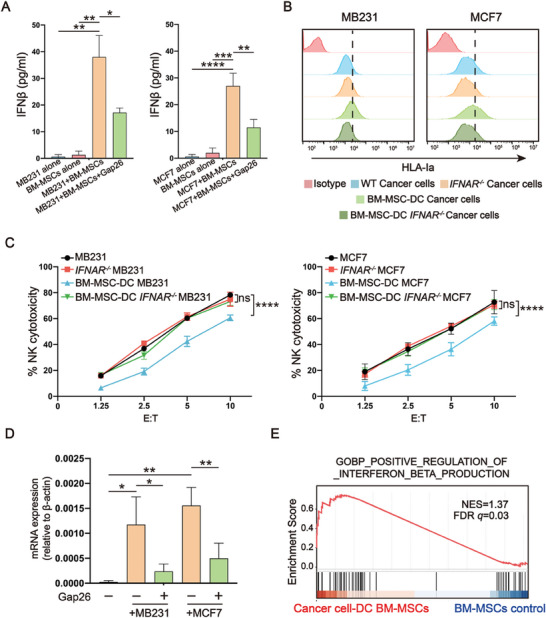
IFNβ production by MSCs increases HLA‐I expression and NK resistance in cancer cells. A) ELISA for IFNβ in cancer cell culture alone, BM‐MSC culture alone, and DC of cancer cells and MSCs and Gap26. N = 3. ^*^ *p* < 0.05, ^**^ *p* < 0.01, ^***^ *p* < 0.001, and ^****^ *p* < 0.0001 per two‐tailed unpaired *t* test. B) Flow cytometry for HLA‐Ia expression on the surface of *IFNAR^−/−^* cancer cells following DC with BM‐MSCs. C) NK assay of *IFNAR^−/−^* cancer cells following DC with BM‐MSCs. N = 3, **p < 0.01 and ****p < 0.0001 per two‐way ANOVA with multiple comparisons. D) qPCR analysis for *IFNB1* in BM‐MSCs following DC with cancer cells and Gap26. N = 3, ^*^ *p* < 0.05 and ^**^ *p* < 0.01 per two‐tailed unpaired *t* test. E) GSEA results showing the enrichment of gene sets related to the positive regulation of IFNβ production in BM‐MSCs following DC with MB231 cells compared to BM‐MSCs without DC.

To identify the ligand for the activated IFN‐I signaling, we measured IFNα and ‐β levels in the conditioned medium from the DC of BM‐MSCs and cancer cells using enzyme‐linked immunosorbent assay (ELISA). The IFNα level remained consistently low across all the tested groups (Figure S4A, Supporting Information), consistent with the previous studies showing that IFNα is produced mainly by immune cells.^[^ ^60^ ^]^ In contrast, the IFNβ level increased in the DC of both MSCs and cancer cells compared to that in the culture of cancer cells or MSCs alone (**Figure** **5**A; Figure S4B, Supporting Information).

To confirm whether the activated IFN‐I signaling is mediated via the IFN‐I receptor IFNAR, we generated *IFNAR1^−/−^* MB231 and MCF7 cells using CRISPR‐Cas9 technology and found that DC with BM‐MSCs could no longer increase HLA‐I expression (Figure 5B) or induce NK resistance in the *IFNAR1^−/−^* cancer cells (Figure 5C) compared to that in WT cancer cells. These data confirm the requirement of IFNAR for the activation of IFN‐I signaling. Finally, to clarify the source of IFNβ, we collected cancer cells and BM‐MSCs from the DC by sorting, and assessed *IFNB1* expression via qPCR. BM‐MSCs, but not cancer cells, had elevated *IFNB1* expression after the DC, which was partially reversed by Gap26 (Figure 5D; Figure S4D,E, Supporting Information). Consistently, GSEA results also revealed the upregulation of IFNβ production‐related gene sets in BM‐MSCs after DC with MB231 cells (Figure 5E and Table S3, Supporting Information). Together, these results suggest that elevated IFNβ in the DC originates from MSCs rather than from cancer cells, which relies on heterocellular gap junction communication, and in turn, stimulates HLA‐I expression in cancer cells.

### 2.6 Activation of STING in MSCs Induces IFNβ Production

**Figure 6:**
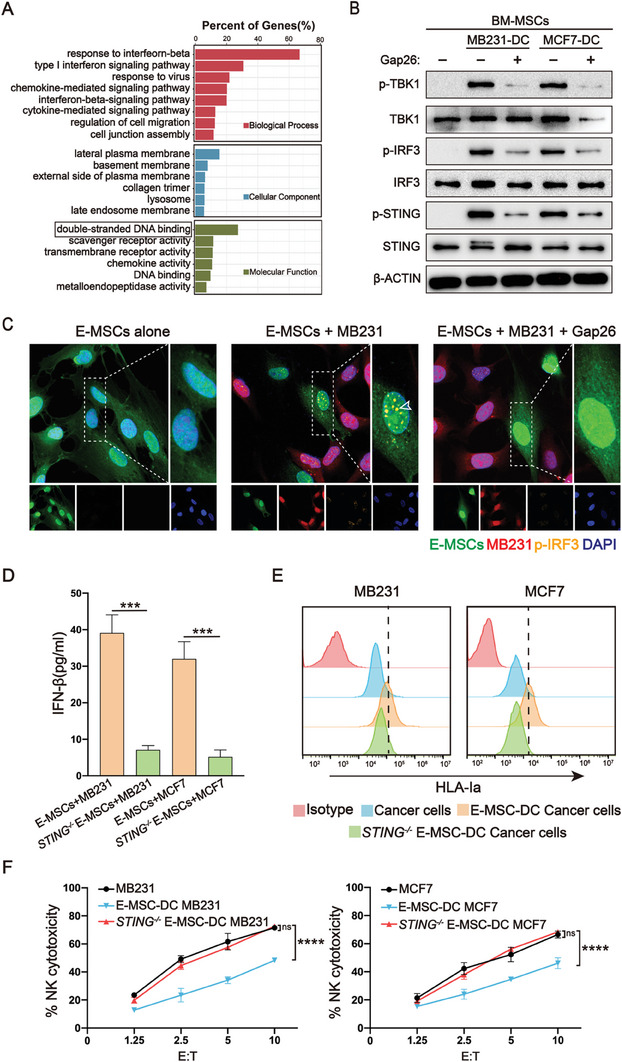
Activation of STING pathway in MSCs induces IFNβ production and NK resistance in cocultured cancer cells. A) GO analysis of RNA‐seq data for E‐MSCs following DC with or without MB231 cells for 3 days. The red, blue, and green bars represent terms enriched for biological processes, cellular components, and molecular functions, respectively. B) Western blotting for phosphorylation of TBK1, IRF3 and STING in BM‐MSCs following DC with cancer cells and Gap26 for 3 days. C) Immunostaining for p‐IRF3 in GFP^+^ E‐MSCs and iRFP^+^ MB231 cells in DC for 3 days. Nuclei were counterstained with DAPI. The white arrowheads denote p‐IRF3 in the nuclei of E‐MSCs. D) ELISA for IFNβ in cancer cell culture alone and DC of cancer cells with WT or *STING* ^−/−^ E‐MSCs for 3 days. N = 3. ^***^ *p* < 0.001 per two‐tailed unpaired *t* test. E) Flow cytometry for HLA‐Ia expression on the surface of cancer cells following DC with WT or *STING* ^−/−^ E‐MSCs for 3 days. F) NK assay for cancer cells following DC with WT or *STING* ^−/−^ E‐MSCs for 3 days. N = 3, ^****^ *p* < 0.01 per two‐way ANOVA with multiple comparisons.

Since increased IFNβ production by MSCs occurred only in the DC with cancer cells, we elucidated how cancer cells stimulate IFNβ production in MSCs via gap junctions. In addition to the the IFN‐I response, genes associated with double‐stranded DNA (dsDNA) binding were also upregulated in BM‐MSCs following DC with MB231 cells (**Figure** **6**A). Cytoplasmic dsDNA can stimulate immune responses.^[^ ^61^ ^]^ In normal cells, the cytoplasm is devoid of DNA.^[^ ^62^ ^]^ However, in cancer cells, a variety of factors such as genome instability, tumor suppressor gene mutation or deletion, and oxidative stress, can result in leakage of dsDNA from the nucleus or mitochondria into the cytoplasm.^[^ ^61^ ^]^ Then, cGAS in the cytoplasm senses dsDNA and generates the second messenger cGAMP, which activates the adaptor protein STING. Activated STING triggers the phosphorylation and activation of TBK1 and IRF3.^[^ ^63^ ^]^ Phosphorylated IRF3 (p‐IRF3) forms dimers and translocates into the nucleus, where it induces the expression of IFNs and other inflammatory cytokines.^[^ ^61^ ^]^ Based on this signaling cascade, we conducted Western blotting on BM‐MSCs from the DC with cancer cells and observed increased levels of phosphorylated (p)‐STING, p‐TBK1, and p‐IRF3 in BM‐MSCs following the DC compared to those without DC (Figure 6B). Nuclear translocation of p‐IRF3 was detected only in E‐MSCs cultured with MB231‐DC but not in E‐MSCs cultured alone, which was also impeded by Gap26 (Figure 6C), suggesting that the gap junction‐dependent activation of STING and its downstream signaling occurred in MSCs following DC with cancer cells.

To elucidate the role of STING activation in MSCs conferring increased NK resistance to cancer cells, we knocked out *STING* in hESCs using CRISPR‐Cas9 (Figure S5A,B, Supporting Information) and then differentiated the *STING* ^−/−^ hESCs into E‐MSCs using a standard protocol^[^ ^64^ ^]^ (Figure S5C, Supporting Information). We conducted DC of cancer cells with WT or *STING* ^−/−^ E‐MSCs and found that *STING^−/−^* E‐MSCs failed to induce IFNβ accumulation in the DC (Figure 6D), concomitant with defects in increasing HLA‐I expression on the surface of cancer cells (Figure 6E) and increasing NK resistance (Figure 6F). Together, these results suggest that activation of STING in MSCs following DC with cancer cells is required to induce IFNβ production in MSCs, which subsequently increases HLA‐I expression and NK resistance in cancer cells.

### 2.7 cGAMP Transferred from Cancer Cells to MSCs Activates STING and Hence IFNβ Production in MSCs

**Figure 7:**
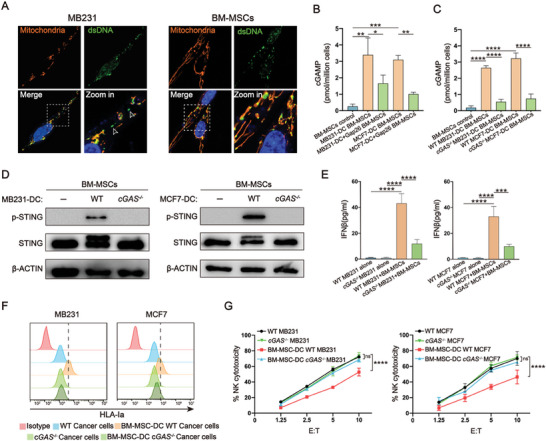
cGAMP transferred from cancer cells to MSCs via gap junctions induces STING activation in MSCs. A) Immunostaining of mitochondria (with MitoTrack, in orange), dsDNA (with PicoGreen, in green), and the nucleus (with Hoechst, in blue). White arrowheads denote dsDNA in the cytosol. B and C) ELISA for cGAMP in the lysates of BM‐MSCs following DC with WT B) or *cGAS^−/−^* C) cancer cells and Gap26. D) Western blotting for p‐STING in BM‐MSCs following DC with WT or *cGAS^−/−^* cancer cells. E) ELISA for IFNβ in the culture of WT or *cGAS^−/−^* cancer cells alone or for that in their DC with BM‐MSCs. F) Flow cytometry for HLA‐Ia expression on the surface of WT or *cGAS^−/−^* cancer cells following DC with BM‐MSCs. Data represent means ± SD. G) NK assay on WT or *cGAS^−/−^* cancer cells following DC with BM‐MSCs. For B, C and E, N = 3, ^*^ *p* < 0.05, ^**^ *p* < 0.01, ^***^ *p* < 0.001, and ^****^ *p* < 0.0001 per two‐tailed unpaired *t* test. For G, N = 3, ^****^ *p* < 0.0001 per two‐way ANOVA with multiple comparisons.

Next, we sought to investigate whether the activation of STING in MSCs was induced by endogenous or exogenous cGAMP. First, we detected dsDNA and mitochondria in cancer cells and MSCs using PicoGreen and MitoTracker, respectively. In addition to the nucleus and mitochondria of cancer cells, a substantial amount of dsDNA was observed in the cytoplasm (**Figure** **7**A). In contrast, in BM‐MSCs, dsDNA was confined to the nucleus and mitochondria (Figure 7A). Moreover, we found that MSCs expressed genes related to the cGAS‐STING pathway at a high level, except for cGAS, whose expression was extremely low (Figure 6B; Figure S6A, Supporting Information), which is consistent with previous study showing that stromal cells such as fibroblasts and MSCs lack the ability to synthesize cGAMP.^[^ ^65^ ^]^

We were interested in understanding how STING was activated in MSCs with such a low level of cGAS. Recent studies have shown that cGAMP in cancer cells can be transferred to neighboring cells through gap junctions.^[^ ^66^, ^67^ ^]^ We hypothesized that cancer cells may transfer cGAMP to surrounding MSCs via gap junctions, which then activates STING in MSCs. To test this hypothesis, we first measured the cGAMP level in both cancer cells and MSCs using ELISA. Indeed, cGAMP was exclusively detected in cancer cells but not in MSCs when they were cultured alone (Figure S6B, Supporting Information). However, after DC of the two cell types, markedly increased cGAMP levels were observed in MSCs, which was reversed by Gap26 (Figure 7B).

To confirm whether the cGAMP transferred from cancer cells is responsible for the activation of STING pathway and consequent production of IFNβ in MSCs, we knocked out *cGAS* in MB231 and MCF7 cells (Figure S6C,D, Supporting Information). Following DC with MSCs, *cGAS^−/−^* cancer cells induced a much lower cGAMP level in MSCs than the WT control cells (Figure 7C) and failed to activate the STING pathway in MSCs (Figure 7D). Concomitantly, MSC‐DC no longer induced IFNβ accumulation (Figure 7E), or increased HLA‐I expression (Figure 7F) and NK resistance (Figure 7G) in *cGAS^−/−^* cancer cells. These data suggest that cGAS in cancer cells is essential for cGAMP synthesis, so cGAMP can be transferred to MSCs to activate STING signaling.

However, it remains to be determined whether cGAMP also induces STING phosphorylation in cancer cells. To answer this question, we tested total STING and p‐STING levels in cancer cells. Although MB231 and MCF7 cancer cells expressed total STING at a basal level (much lower in MCF7 cells than in MB231 cells), p‐STING was undetectable in both cancer cells regardless of DC with MSCs or without MSCs (Figure S6E, Supporting Information). These results are consistent with the previous studies showing that tumor cells can develop multiple strategies to suppress the intrinsic cGAS‐STING signaling, e.g., downregulating STING expression or reducing sensitivity to dsDNA or cGAMP.^[^ ^68^, ^69^ ^]^ Thus, it is challenging to induce STING activation and subsequent IFNβ production in cancer cells when the cGAMP level remains below a certain threshold.^[^ ^70^, ^71^ ^]^

### 2.8 High IFN‐I Signaling and HLA‐I Levels are Correlated in Human Tumors and Predict a Poor Patient Outcomes

**Figure 8:**
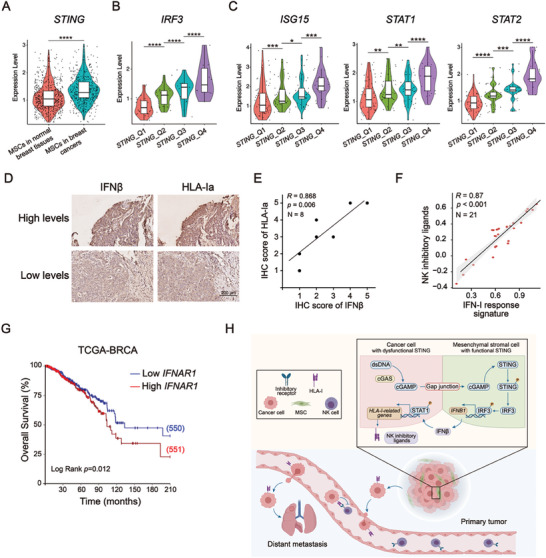
IFN signaling and HLA‐I levels are correlated in human breast cancer. A) Violin plots showing the expression of *STING* in MSCs from normal breast tissues and breast cancer tissues. B and C) Violin plots showing the expression of *IRF3* and IFNβ signaling‐related genes (*ISG*, *STAT1* and *STAT2*) in breast cancer MSCs. For A, B & C, Mann‒Whitney two‐sided test was used to test the significance of differences in gene expression between two groups. ^*^ *p* < 0.05, ^**^ *p* < 0.01, ^***^ *p* < 0.001 and ^****^ *p* < 0.0001. D) Immunohistochemistry (IHC) for IFNβ and HLA‐Ia expression in human breast cancer samples with two representative areas displayed. Scale bars: 200 µm. E) Quantification of the intensity of staining per sample from D. The intensity was recorded via ImageJ and presented as an IHC score. The correlation between the IHC scores of IFNβ and HLA‐Ia in 8 (two overlapping) detected human breast cancer samples is displayed in a graph with coefficient *R* and *P* values (right). Each dot represents one sample. F) Correlation analysis of NK inhibitory ligands and IFN‐I response signature in CTCs collected from breast cancer patients. G) Kaplan‒Meier plot of overall survival curves for breast cancer patients with low or high expression level of *IFNAR1* expression. H) Schematic for MSC‐induced CTC resistance to NK cells via the intercellular cGAS‐STING‐IFNβ‐HLA axis.

To determine the clinical relevance of our findings above, we further analyzed scRNA‐seq data on MSCs in the clinical samples described above (Figure 1B)^[^ ^23^ ^]^ and found that *STING* was upregulated in breast cancer MSCs compared to that in MSCs from normal breast tissues (**Figure** **8**A). We divided that breast cancer MSCs into four groups, Q1‐Q4, based on the expression level of *STING* (Figure S7A, Supporting Information). A positive correlation was found between the expression of *STING* and its target gene *IRF3* in breast cancer MSCs (Figure 8B), indicating the activation of STING signaling in breast cancer MSCs. Moreover, *STING* expression was also positively correlated with the expression of the IFN‐I signaling genes *ISG15*, *STAT1* and *STAT2* in breast cancer MSCs (Figure 8C). These data from clinical samples support the correlation between activated STING signaling and IFN‐I signaling in the TME.

To evaluate the clinical relevance of IFN‐I signaling and cancer cell resistance to NK cells, we measured the levels of IFNβ and HLA‐I expression in human breast tumor biopsies via immunohistochemistry. The results indicated a positive correlation between HLA‐Ia expression and IFNβ levels in the primary tumors, yet it was difficult to determine the origin of IFNβ in the samples (Figure 8D,E). Next, we selected the top 20 upregulated genes in MB231 cells with prior MSC‐DC compared to control cells without MSC‐DC and designated the gene set as the IFN‐I response signature (Figure S7B and Table S3, Supporting Information). Transcriptionally, the gene set was positively correlated with the expression of NK inhibitory ligands in primary breast tumors^[^ ^72^ ^]^ (Figure S7C, Supporting Information). A similar correlation was observed in CTCs obtained from breast cancer patients based on our analysis of RNA‐seq data^[^ ^74^ ^]^ (Figure 8F), suggesting that IFN‐I signaling in the TME increases the NK resistance of CTCs before they exit from a primary tumor. Furthermore, we conducted Kaplan‒Meier plotter analysis based on the TCGA‐BRCA database^[^ ^75^ ^]^ to determine the association between IFN‐I signaling and the overall survival of cancer patients. Higher *IFNAR1* expression was associated with lower survival in breast cancer patients (Figure 8G) and patients with other cancer types including lung squamous cell carcinoma and pancreatic adenocarcinoma (Figure S7D, Supporting Information).

To summarize all our findings above, we propose a working model composed of five intercellular chain reactions: 1) cytoplasmic dsDNA in cancer cells is sensed by cGAS, which induces the synthesis of cGAMP; 2) cGAMP is transferred from cancer cells into MSCs via gap junctions; 3) transferred cGAMP induces STING activation and subsequent IFNβ production in MSCs; 4) IFNβ from MSCs binds IFNAR on cancer cells to trigger an IFN‐I response, increasing HLA‐I expression in cancer cells; 5) elevated HLA‐I on cancer cells protects the cells from NK cell‐mediated cytotoxicity; and 6) increased NK‐resistance promotes CTC seeding and metastasis formation in the distant organs (Figure 8H).

## 3 Discussion

In this study we demonstrated that MSCs empowered CTCs with increased resistance to NK cell‐mediated killing, hence, promoting their survival in the circulation and tumor metastasis in the lungs of the host. MSCs acquire these abilities by interacting with cancer cells during coculture. Cancer cells delivere cGAMP via the gap junctions to the neighboring MSCs. Upon entry into MSCs, cGAMP activates STING to increase *IFNβ* expression in the MSCs. IFNβ secreted by MSCs binds to IFNβ receptors on cancer cells to activate the *HLA‐I* gene expression, resulting in an increase in HLA‐I molecules on the surface of tumor cells, which become resistant to NK cell‐mediated killing. Our findings thus reveal an overlooked tumor‐promoting effect of MSCs in the TME by increasing the expression of NK inhibitory ligands in CTCs via an intercellular cGAS‐STING‐IFNβ‐HLA signaling loop, which helps CTCs evade NK cell surveillance in the blood.

The immune system, including NK and T cells, plays a key role in controlling metastatic disease and destroying metastatic colonies.^[^ ^76^ ^]^ It has been reported that NK and T cells preferentially exert distinct roles in immunosurveillance at different stages of tumor progression.^[^ ^77^ ^]^ To evade immunosurveillance, cancer cells dynamically change HLA‐I levels to alter their susceptibility to T cells or NK cells.^[^ ^78^ ^]^ For example, in established solid tumors, infiltrating NK cells rapidly lose their antitumor functions;^[^ ^79^ ^]^ thus, cancer cells mainly need to evade T‐cell immunity via various mechanisms, including downregulation of HLA‐I expression.^[^ ^80^ ^]^ CTCs travel in the circulation transiently, so successful immunosurveillance requires rapid and direct immunocytes responses.^[^ ^81^ ^]^ Compared with T cells and other immunocytes, NK cells predominantly control CTCs.^[^ ^82^ ^]^ It has been known that CTCs upregulate HLA‐I levels to escape NK cytotoxicity via a variety of mechanisms.^[^ ^82^, ^83^ ^]^ In this study, we revealed a novel mechanism by which CTCs to evade NK cell surveillance, i.e., the upregulation of HLA‐I expression via an intercellular cGAS‐STING‐IFNβ‐HLA signaling loop.

The cytosolic DNA‐sensing cGAS‐STING pathway has emerged as a fundamental mechanism driving inflammation‐mediated tumorigenesis.^[^ ^84^, ^85^ ^]^ Unlike normal cells, cancer cells often have abundant dsDNA in their cytosol, which is mainly attributed to chromosomal instability (CIN).^[^ ^86^, ^87^ ^]^ Accordingly, CIN‐mediated chronic activation of cGAS‐STING and downstream noncanonical NF‐κB signaling promote cellular invasion and metastasis characterized by epithelial‐mesenchymal transition and inflammation.^[^ ^88^ ^]^ Activation of cGAS‐STING pathway also triggers the production of IFN‐I,^[^ ^61^ ^]^ which influences tumor progression in all phases. In early neoplastic progression, STING/IFN‐I responses induce tumor cell apoptosis, inhibit tumor cell proliferation and metastasis, and upregulate HLA‐I expression in tumor cells to facilitate adaptive immunity against tumor cells.^[^ ^89^ ^]^ IFN‐I also promotes innate immunity by inducing NK cell maturation and activation and increasing NK cell cytotoxicity, which is critical for immunosurveillance during early tumorigenesis.^[^ ^90^ ^]^ However, cancer cells have developed strategies to circumvent these suppressive effects by driving protumorigenic programs. Persistent and weak STING/IFN‐I pathway cultivates the immunosuppressive tumor microenvironment by recruiting myeloid cells via CCR2.^[^ ^91^ ^]^ Moreover, IFN‐I is implicated in upregulating the expression of immune checkpoints, such as PD‐L1, in tumor cells, increasing their resistance to immunotherapies.^[^ ^92^ ^]^

Dysfunction STING signaling often occurs in human primary cancer cells and cancer cell lines, resulting in reduced production of DNA damage‐related cytokines, including IFN‐I.^[^ ^93^ ^]^ This deficiency was also observed in MB231 and MCF7 cancer cells cultured alone in our study. Interestingly, the transfer of cGAMP to neighboring MSCs, which possess normal STING functions, can relay the cGAS‐STING signaling. In this study, we elucidated a novel effect of cGAS‐STING on shaping the chronic inflammatory microenvironment, which underscores the profound impact of the stromal context.

cGAMP can diffuse to surrounding cells through various mechanisms and induce bystander activation of STING signaling. For example, cell death causes the release of cGAMP into the extracellular environment. The released cGAMP then enters neighboring cells, most likely via SLC19A1, a transmembrane transporter.^[^ ^94^, ^95^ ^]^ Gap junctions are also considered as important channels for intercellular transfer of cGAMP.^[^ ^66^ ^]^ This horizontal transfer of cGAMP not only amplifies the local detection of cytosolic dsDNA, but also modulates the TME. For example, astrocytes receive cGAMP from tumor cells through gap junctions and produce IFNα and tumor necrosis factor; as a result, these cytokines in turn enhance the survival and metastasis of the tumor cells.^[^ ^67^ ^]^ In contrast, dendritic cells are primary immune responders to tumor‐derived cGAMP, which augments the antitumor functions of NK cells and T cells.^[^ ^96^, ^97^ ^]^ Therefore, the bystander activation of STING pathway in surrounding cells elicits either pro‐ or antitumorigenic effects, depending on the cell type and context.

The cGAS‐STING pathway is involved in different parts of the cancer‐immunity cycle to promote or suppress antitumor immune responses. Combination therapy targeting cGAS‐STING has made certain progress in both preclinical and clinical trials, but the development of optimal treatment regimens remains challenging.^[^ ^98^ ^]^ Nevertheless, the findings from this study are encouraging, as they provide novel insights into the NK resistance in CTCs and may lead to new strategies to eliminate CTCs and prevent tumor metastasis.

## 4 Experimental Section

### Ethics Statement

This study strictly followed the International Society for Stem Cell Research guidelines for studies on hESCs. Sections of human breast cancer patients were obtained from C.D. under ethics protocol #BSERE20‐APP006‐FHS, which was approved by the University of Macau Subpanel on Biomedical Science and Engineering Research Ethics. The animal experiments in this study were governed by the animal use protocols # UMARE‐016‐2020 (mouse lung metastasis experiment) and #UMARE‐032‐2016 (zebrafish experiment), which were approved by the University of Macau Animal Research Ethics Subpanel.

### Cell Lines and Ethics Regulations

All experiments were conducted in accordance with the National Institutes of Health Guidelines on Human Stem Cell Research. The hESC line Envy (GFP^+^),^[^ ^34^ ^]^ was maintained in a monolayer on Matrigel (Corning, 354232) in mTeSR medium (Stemcell, #85851). hESCs were differentiated into MSCs via an intermediate trophoblast step using a 3D method, as we reported previously.^[^ ^99^ ^]^ BM‐MSCs and Ad‐MSCs were derived from human bone marrow aspirates^[^ ^100^ ^]^ and adipose tissues,^[^ ^101^ ^]^ respectively, from healthy adults using protocols approved by the University of Macau Panel of Research Ethics.^[^ ^100^ ^]^ MSCs were cultured in α‐MEM medium (Thermo, #12571063) supplemented with 20% fetal bovine serum (FBS) (Thermo, #A31608), 1% non‐essential amino acids (Gibco, #11140‐068) and 1% L‐glutamine (Gibco, #2036886) at 37 °C with 5% CO_2_. MSCs were passaged every 5–7 days and characterized for typical MSC markers via flow cytometry. All the experiments used MSCs within 10 passages.

The breast cancer cell lines MB231 and MCF7, the colon cancer cell line LoVo, and the hepatocarcinoma cell line HepG2 were cultured in a high‐glucose DMEM medium (Thermo, #11965118) supplemented with 10% FBS at 37 °C with 5% CO_2_.^[^ ^102^, ^103^ ^]^ The cells were labeled with iRFP or GFP via lentiviral transduction and passaged every 3–4 days. All the cell lines were confirmed to be free of mycoplasma contamination.

### Coculture of MSCs and Organoids

Dissociated cell clusters were resuspended in 50% cold Matrigel (Corning, #356234) and gently dispersed as 25 µL droplets into prewarmed 6‐well plates. Subsequently, the droplets were solidified by incubating them in a 37 °C, 5% CO2 incubator for 30 min. Then, 2.5 mL of the previously established organoid culture medium^[^ ^33^ ^]^ was added to each well and replenished every 2–3 days. After 7 days of culture, the Matrigel droplets were melted using precooled organoid culture medium, and the larger‐sized organoids (40–100 µm) were collected and fluorescently stained with TO‐PRO‐3 (1:3000; Thermo Fisher). Then the stained organoids were mixed with MSCs at a 1:1 ratio in cell numbers and cultured in the organoid culture medium for 72 h for subsequent functional experiments.

### Reagents

Gap26 (#A1044) was purchased from APExBIO Technology (USA), and MitoTracker Red CM‐H2Xros (#M7513) and Hoechst 33 342 solution (#62 249) were purchased from Thermo Fisher (USA). PicoGreen dsDNA reagent (#MF0784) was purchased from MKBio (China).

### Preparation of Artificial Antigen‐Presenting Cells (aAPCs) and Activation and Expansion of Primary NK Cells

To establish aAPCs, we cloned human interleukin‐21 (hIL‐21) into a vector that contained a human immunoglobulin G1 (IgG1) hinge, CD8 transmembrane domain and intracellular domain, 4‐1BB intracellular domain, and CD3ζ intracellular domain as previously reported^[^ ^104^, ^105^ ^]^ and sequenced all the components (Table S4, Supporting Information). K562 human leukemia cells were transduced with the hIL‐21‐expressing lentiviral vector, and hIL‐21^+^ cells were sorted with FACSAria III (BD Biosciences) using phycoerythrin (PE)‐conjugated mouse anti‐hIL‐21 antibody (Invitrogen, clone 3A3‐N2) and used as aAPCs. aAPCs were cultured in RPMI 1640 (Thermo, #11 875 093) supplemented with 10% FBS at 37 °C with 5% CO_2_. Before being used, aAPCs were irradiated at a dose of 80 Gy, washed with PBS, and then used as feeder cells.

For NK cell activation and expansion, peripheral blood monocytes (PBMCs) were isolated from buffy coats using Lymphoprep (Stemcell, #0 7851), and cultured with the irradiated aAPC feeder cells at a ratio of 1:2 in RPMI 1640 media supplemented with 10% FBS, 200 U mL^−1^ IL‐2 (PeproTech, #200‐02), and 5 ng mL^−1^ IL‐15 (PeproTech, #200‐15). The medium was were refreshed every 3–4 days. The percentage of NK cells among the PBMCs was determined as the percentage of CD3^+^/CD56^+^ cells per flow cytometry.^[^ ^106^ ^]^

### In Vitro NK Cytotoxicity Assay

As previously described,^[^ ^36^ ^]^ ten thousand target cells were labeled with Calcein‐AM (eBioscience, #65‐0853‐78, 5 µM) and then seeded in 100 µl per well in a flat‐bottom 96‐well plate. Then, 100 µl of human primary NK cells (effector cells) was added in 100 µl to mix with the presided target cells at various E/T ratios and the cells were incubated for 4 h at 37 °C with 5% CO2. All groups were set up in triplicate. Target cells without NK cells were set as spontaneous release, and target cells lysed with 1% Triton X‐100 (Sigma, #93 443) were set as the maximum release. Then, 100 µL of supernatant was collected from each well and measured using a Victor X5 microplate reader (PerkinElmer) with an excitation filter at 485 nm and an emission filter at 535 nm. The percent lysis was calculated using the following formula:

(1) $$\begin{array}{ccc} \mathrm{Specific}\;\mathrm{Lysis}\;\%\; & = & \frac{\mathrm{Test}\;\mathrm{release}-\mathrm{Target}\;\mathrm{spontaneous}\;\mathrm{release}}{\mathrm{Maximum}\;\mathrm{release}-\mathrm{Target}\;\mathrm{spontaneous}\;\mathrm{release}}\\ & & \;\times 100 \end{array}$$

### NK Cytotoxicity Assay in Animals and Ethics Regulations

For NK cytotoxicity assay in zebrafish, Tg(*fli1*: EGFP) zebrafish embryos at 48 h post fertilization were subjected to perform the microinjection as described previously.^[^ ^46^ ^]^ After coculture with BM‐MSCs for 3 days, the 231‐C3 cells were sorted and resuspended in PBS. For single cell injection, the final concentration of 231‐C3 cells was adjusted to 2.5 × 10^4^ cells µL^−1^. For coinjection, the final concentration of 231‐C3 cells together with the human NK cell line, NK‐92MI cells, which had been transfected with the red fluorescent protein tandem dimer Tomato (NK‐92MI‐tdTomato),^[^ ^46^ ^]^ was adjusted to 5 × 10^4^ cells µL^−1^ at a ratio of 1:1. Then, 231‐C3 cells alone (≈100 cells) or together with NK‐92MI‐tdTomato (≈200 cells at a ratio of 1:1) were microinjected into the common cardinal vein of zebrafish using an MPPI‐3 pressure injector (Applied Scientific Instrumentation). After injection, the zebrafish were maintained in zebrafish incubating water for 8 h. Fluorescence images were taken at 8 h after the injection using a Carl Zeiss LSM 880 confocal laser scanning microscope. The 231‐C3 cells were excited using a 458 nm laser. Emissions at 460–500 nm for the cyan fluorescent protein (CFP) signal and 520–550 nm for the yellow fluorescent protein (YFP) signal were collected at the same time and merged to construct FRET images. After merging, live cells emit green fluorescence due to the energy transfer from CFP to YFP. In apoptotic cells, activated caspase‐3 cleaves the sensor C3 protein at the DEVD site to abrogate the FRET effect. Thus, apoptotic cancer cells emit blue fluorescence. In addition, the vessels of Tg (*fli1*: EGFP) zebrafish can be imaged using 458‐nm laser and excitation with a 488 nm laser. NK‐92MI‐tdTomato cells were imaged using a 561 nm laser, and emissions between 578 and 696 nm were measured. Then, z‐stack images were taken to capture the 3D distribution of cancer cells and NK cells. ImageJ software was used to process the projection of the z‐stack.

For the NK cytotoxicity assay in mice, NOD/SICD mice (8 weeks old) were intravenously injected with 10^6^ GFP‐labeled MB231 cells/mouse. Primary human NK cells (10^7^ cells/mouse) were intravenously injected 0.5 h before cancer cell injection. To elongate the lifespan of the injected NK cells in vivo, all mice received an intraperitoneal (i.p.) injection of IL‐2 (50 000 U per mouse) and IL‐15 (10 ng per mouse) at 1 h before NK cell injection.^[^ ^105^ ^]^ At 28 days postinjection, the mice were sacrificed, and their lungs were collected. Tumor nodules on the surface of the lungs (representing pulmonary metastasis) were imaged using an Olympus MVX10 macrozoom florescence microscope.

### Genome Editing

The CRISPR/Cas9 system^[^ ^107^ ^]^ was used to knock out the *B2M*, *IFNAR1*, *cGAS*, and *STING* genes. The plasmid pSpCas9(BB)−2A‐GFP (PX458) was a gift from Feng Zhang (Addgene plasmid #48 138). The guide sequences were designed using the online CRISPR design tool (http://crispor.tefor.net/crispor.py). The sgRNA sequences (Table S2, Supporting Information) were each inserted into the *Bbsl* site of PX458 to generate the targeting plasmids PX458‐sgB2M (for *B2M*), PX458‐sgIFNAR1 (for *IFNAR1*), PX458‐sgcGAS (for *cGAS*), and PX458‐sgSTING (for *STING*). MB231 and MCF7 cells were transfected with PX458‐sgB2M, PX458‐sgIFNAR1 and PX458‐sgcGAS using Lipofectamine 3000 reagent (Invitrogen, #L3000015). Envy hESCs were transfected with PX458‐sgSTING using Lipofectamine Stem Transfection Reagent (Invitrogen, #STEM00015). GFP^+^ cells were enriched using FACSAria III (BD Biosciences). Single‐cell clones were obtained using the IncuCyte Live Cell Analysis Imaging System (Sartorius) and verified for knockout of the target genes via genomic PCR using specific primers (Table S2, Supporting Information).

### RNA‐Seq Analysis

MB231 cells following DC with MSCs for various durations were collected. MB231 cells without DC were used as a control. Each group contained three replicates. RNA was extracted from cell pellets using the Qiagen RNeasy Mini Kit according to the manufacturer's instructions. RNA samples were sent to Genewiz (China) for RNA‐seq analysis. The adapter sequences were removed by the Cutadapt software (V1.9.1) and quality control (QC) was assessed using the FastQC software (V0.10.1). Sequence reads were aligned to the human (*homo sapiens*) reference genome (hg19) using the HISAT2 software (V2.0.1) and gene counts were calculated with the HTSEQ software (V0.6) separately. The raw count data were imported to R language (version 4.2.1) for further testing.

Fragments Per Kilobase of transcript per Million mapped reads (FPKM) was normalized for differential gene expression analysis using EdgeR (V3.4.6) and heatmap visualization. Differentially expressed genes (DEGs) were defined based on the p.adj < 0.05. Gene ontology (GO) enrichment analysis was performed by using the GOSeq package (V1.34.1). GSEA was performed to screen biological pathways with the hallmark Hecker IFNβ target gene set and positive regulation of IFNβ production gene set based on the curated gene sets and ontology gene sets, respectively, in the GSEA molecular signature database.

The TCGA data on breast cancer gene expression were obtained from the Genomic Data Commons Data Portal.^[^ ^75^ ^]^ The RNA‐seq data on CTCs from 21 biospecimens of 19 breast cancer patients were reported by Ring et al.^[^ ^108^ ^]^ Single sample GSEA was performed to test the transcriptional correlation between IFN‐I signaling and NK inhibitory ligands in primary tumors and CTCs from the breast tumor patients.

### scRNA‐Seq Analysis

The scRNA‐seq data of 69 distinct surgical tissue specimens from 55 breast cancer patients versus normal breast tissues from 18 women with reduction mammoplasties were reported by Pal et al.^[^ ^23^ ^]^ The expression sparse matrix containing cell type annotations was generated using the Seurat (V4.3.0). First, the raw count data were normalized using the LogNormalize method. Next, we utilized the FindVariableFeatures function to calculate the top 2000 highly variable genes and the principal component (PC) calculations were conducted using the top 2000 highly variable genes. The Harmony algorithm was used for single cell integration and batch effect correction. The uniform manifold approximation and projection (UMAP) embedding was generated using the calculated PCs. Lymphocytes (CD3D, CD3E and CD3G), myeloid cells (CD68), endothelial cells (PECAM1 and VWF), fibroblasts (PDGFRA, but negative for ALDH1A1, HLF4 and LEPR), MSCs (PDGFRA, ALDH1A1, HLF4 and LEPR), and epithelial cells (EPCAM and KET19) were identified. We then repeated the data processing steps, including normalization, PC calculation, and Harmony integration. Finally, CellChat^[^ ^28^ ^]^ was utilized for the cell‒cell communication analysis.

### Distinguishing Cancerous Epithelial Cells from Normal Breast Epithelial Cells

The CNV signal for individual cells was estimated using the inferCNV method v.0.99.7 with a 100‐gene sliding window. Endothelial cells were used to define the reference cell‐inferred copy number profiles. Epithelial cells were used for the observations. According to the process previously described by Neftel et al.,^[^ ^109^ ^]^ cancerous epithelial cells were separated from normal breast epithelial cells.

### Flow Cytometry

Cells were harvested using 0.05% trypsin‐EDTA to obtain single‐cell suspensions. For cell surface staining, the cells were incubated with primary antibodies (Table S1, Supporting Information) for 30 min on ice, followed by incubation with the corresponding secondary antibodies (Table S1, Supporting Information) for another 30 min on ice. The data were collected using the Cytoflex cytometer (Beckman Coulter) and analyzed using the FlowJo software.

### Real‐Time qPCR

Total RNA was extracted using TRIzol reagent (Thermo, #15 596 026) according to the manufacturer's instructions. Reverse transcription was performed using the PrimeScript RT Reagent Kit with gDNA Eraser (Takara, #RR047A). qPCR was performed using iTaq Universal SYBR Green Supermix (Bio‐Rad, #1 725 124) and gene‐specific primers (Table S2, Supporting Information) as previously described.^[^ ^106^ ^]^

### Western Blotting

Cells were lysed in the radioimmunoprecipitation assay (RIPA) buffer (Thermo, #89 900) supplemented with proteinase inhibitor (Thermo, #87 786). After denaturation on a heat‐block, 50 µg of protein per sample per lane was separated on a 10% SDS‐PAGE gel (Bio‐Rad, #1 610 173) and then transferred to a PVDF membrane (Bio‐Rad, 1 620 177). The membrane was blocked with a 5% blocking buffer and then incubated with a primary antibody overnight at 4 °C, followed by incubation with the corresponding secondary antibodies (Table S1, Supporting Information). The blots were finally incubated in Clarity Western ECL Substrate (Bio‐Rad, #1 705 061) and visualized using a ChemiDoc Imager (Bio‐Rad, #170‐8370).

### Immunofluorescence (IF)

Monolayer cultured cells were washed with PBS three times and fixed with 4% paraformaldehyde (PFA). After treatment with 0.1% Triton X‐100 and 5% BSA, the samples were stained with the corresponding antibodies (Table S1, Supporting Information). Images were taken using the Carl Zeiss LSM 710 confocal fluorescent microscope.

### Immunohistochemistry (IHC)

Resected tumors and organs were fixed in 4% paraformaldehyde (PFA), then dehydrated with xylene and embedded in paraffin. The samples were cut into 8 µm slices, and the slices were transferred to positive adhesion glass slides and hydrated by ethanol. After antigen retrieval in citric acid buffer in a microwave, samples were stained with corresponding antibodies (Table S1, Supporting Information). Then samples were applied DAB substrate solution and Hematoxylin. Slide scans were taken using the Hammatsu Digital Slide Scanner NanoZoomer S60. The IHC score per sample was calculated based on the intensity of staining recorded using ImageJ.^[^ ^110^ ^]^ The scores were rated from 1 to 5 based on the staining intensity from weak to strong.

### Statistical Analysis

All data are presented as the mean ± standard deviation (SD). All in vitro cell experiments included three biological replicates. All statistical analyses were performed using GraphPad Prism software. *P* < 0.05 was considered to indicate a statistically significant difference. Two‐way ANOVA tests were used to analyze the data derived from the NK cytotoxicity assay. Mann‒Whitney two‐sided tests were used for nonparametric data. A log‐rank test was used for the Kaplan‒Meier curves comparing the different patient groups. The experimental sample numbers (n) and *P* values are shown in the figures, figure legends, and results section.

## Conflict of Interest

R.X. is a founder of ImStem Biotechnology, Inc., a stem cell company. The other authors declare no competing financial interests.

## Author Contributions

R.X. and Y.Y. conceived of and designed the study. Y.Y., G.Q., H.Y., H.J., D.Z., Z.Z., and S.Y. performed the experiments and analyzed all the data. Y.Y., K.Q.L., C.D., and R.X. wrote the manuscript. R.X. gave the final approval of the manuscript.

## Supporting information

Supporting Information

## Data Availability

The data that support the findings of this study are available from the corresponding author upon reasonable request.
