# Supplementary material for: Mesenchymal Stromal Cells Increase the Natural Killer Resistance of Circulating Tumor Cells via Intercellular Signaling of cGAS‐STING‐IFNβ‐HLA
Source: Adv Sci (Weinh). 2024 Apr 18;11(21):2400888. doi: 10.1002/advs.202400888 (PMC11151078; doi:10.1002/advs.202400888)
Supplement: Supplementary file 1 — Supporting Information [file ADVS-11-2400888-s001.pdf]

ADVANCED SCIENCE
Open Access

Supporting Information

for *Adv. Sci.*, DOI 10.1002/advs.202400888

Mesenchymal Stromal Cells Increase the Natural Killer Resistance of Circulating Tumor Cells via Intercellular Signaling of cGAS-STING-IFNβ-HLA

*Ye Yi, Guihui Qin, Hongmei Yang, Hao Jia, Qibing Zeng, Dejin Zheng, Sen Ye, Zhiming Zhang, Tzu-Ming Liu, Kathy Qian Luo, Chu-Xia Deng and Ren-He Xu**

Supporting Information

Mesenchymal stromal cells increase the natural killer resistance of circulating tumor cells via intercellular signaling of cGAS-STING-IFNβ-HLA

Ye Yi, Guihui Qin, Hongmei Yang, Hao Jia, Qibing Zeng, Dejin Zheng, Sen Ye, Zhiming Zhang, Tzu-Ming Liu, Kathy Qian Luo, Chu-Xia Deng, Ren-He Xu[*]

[*]Corresponding author: Ren-He Xu, Faculty of Health Sciences, University of Macau, Taipa, Macau, China. Email address: renhexu@um.edu.mo.

Table S1. List of antibodies used in this study

Antibody name	Company	Catalog #	Application
p-TBK1	CST	5483	WB (1:1000)
TBK1	CST	3504	WB (1:1000)
p-IRF3	CST	4947	WB (1:1000), IF (1:100)
IRF3	CST	4302	WB (1:1000)
cGAS	CST	79978	WB (1:1000)
p-STING	CST	50907	WB (1:1000)
STING	CST	13647	WB (1:1000)
p-STAT1	CST	9167L	WB (1:1000)
STAT1	SANTA CRUZ	sc-346	WB (1:1000)
β-Actin	SANTA CRUZ	SC-130301	WB (1:1000)
HLA-ABC	eBioscience	11-9983-42	Flow (1:100)
HLA-ABC	Beytime	AG2146	IHC (1:100)
IL-21	Biolegend	513003	Flow (1:100)
IFNβ	Invitrogen	16-9978-85	Neutralization (1:200)

IFNβ	Invitrogen	PA5-20390	IHC (1:100)
FITC Isotype control	BD Biosciences	555742	Flow (1:100)
PE Isotype control	BD Biosciences	559320	Flow (1:100)
HRP, anti-Rabbit IgG	Invitrogen	G-21234	WB (1:10000)
HRP, anti-mouse IgG	Invitrogen	G-20140	WB (10000)
Alex Fluor 488, Anti-Rabbit IgG	Invitrogen	A-21207	IF (1:100)
Human MSC analysis kit	BD Biosciences	562245	
Human IFNβ ELISA kit	R&D systems	DIFNB0	
Human IFNα ELISA kit	Neobioscience	EHC144a.48	
cyclic GAMP ELISA kit	Invitrogen	17174853	

Note: CST: Cell Signaling Technology. WB: Western blotting. IF: Immunostaining. IHC: Immunohistochemistry. Flow: Flow cytometry.

Table S2. List of oligonucleotides used in this study

**Primer name**	**Sequences (5'-3')**
B2M-sgRNA-F	CACCGCGCGAGCACAGCTAAGGCCA
B2M-sgRNA-R	AAACTGGCCTTAGCTGTGCTCGCGC
cGAS-sgRNA-F	CACCGGACTCGGTGGGATCCATCG

cGAS-sgRNA-R	AAACCGATGGATCCCACCGAGTCC
STING-sgRNA-F	CACCGGAGCACACTCTCCGGTACC
STING-sgRNA-R	AAACGGTACCGGAGAGTGTGCTCC
IFNAR1-sgRNA-F	CACCGAAGCAGCACTACTTACGTCA
IFNAR1-sgRNA-R	AAACTGACGTAAGTAGTGCTGCTTc
B2M-T7-F	GGGAGGAACTTCTTGGCACA
B2M-T7-R	GACGCTTATCGACGCCCTAA
cGAS-T7-F	CGGATTGCCTGGAGAGTTAG
cGAS-T7-R	GAAGGTAGGGACTGCGG
STING-T7-F	CTGAGACAGGAGCTTTGG
STING-T7-R	CCTCCATCAAGGACACC
IFNAR1-T7-F	GATTTTTTTGCAGCTCAG
IFNAR1-T7-R	GATTACAGGCGTGGGCCAC
IFNβ-F	TCCCAGGAACTCAATGAAGG
IFNβ-R	GTGTCGCAATGGAGTGTTGT
β-Actin-F	CCTCGCCTTTGCCGA
β-Actin-R	TGGTGCCTGGGGCG

Table S3. The composion of gene set and signature genes related to STAR Methods.

**Signature or gene set name**	**Gene name**
**IFN-I response signature**	AF1; MX1; IFIT1; IRF7; OAS1; OAS3; OAS2; IFIT3; EGR1; IFI6; OASL; IFITM3; IFI27; MX2; USP18; IRF7; IFIT1; IFITM1; ISG15; IFIH1; IRF5; TYK2; IFI6; IFIT3; IFI35; IRF9; IFITM2; IRF1; SAMHD1; RSAD2; MT2A

**IFN-I target gene set**	APOBEC3A; APOBEC3B; ARG1; C1QA; C1QB; C1QC; C3AR1; CABP5; CCL2; CCL8; CD163; CHI3L1; CLU; CMPK2; CMTM2; CMTM5; CXCL10; CYP4F3; DDX58; DDX60; DHX58; DTX3L; EGR1; EGR2; EIF2AK2; EPSTI1; FCER1A; FFAR2; G0S2; GBP1; GGTA1; GP9; GZMB; HERC5; HERC6; HES4; IFI27; IFI35; IFI44; IFI44L; IFI6; IFIT1; IFIT2; IFIT3; IFIT5; IL8; IRF7; ISG15; ITGA2B; JUP; KCNJ15; KLRD1; LAMP3; LAP3; LGALS3BP; LILRA3; LOC391020; LY6E; MGAM; MMP25; MMP9; MS4A4A; MX1; MX2; MYL9; OAS1; OAS2; OAS3; OASL; PARP12; PARP9; PDZK1IP1; PLSCR1; PNPT1; PROS1; RPS23; RSAD2; RTP4; SAMD9; SAMD9L; SCO2; SERPING1; SH3BGRL2; SIGLEC1; SPON2; STAP1; STAT1; TCL1A; TMEM140; TNFAIP6; TRBV27; TREML1; TYMP; XAF1; ZBP1.
**Positive regulation of IFNβ production gene set**	ARRDC4; D1PAS1; DDX3X; DHX9; FLOT1; HMGB1; HMGB2; HSP90AA1; IFIH1; IFNAR1; IRF1; IRF3; IRF7; ISG15; MAVS; OAS1A; OAS1B; OAS1C; OAS1D; OAS1E; OAS1F; OAS1G; OAS1H; OAS2; OAS3; POLR2A; POLR3B; POLR3C; POLR3D; POLR3F; PLLR3G; PTPN11; PTPN22; RIGI; RIOK3; RNF135; STING; TBK1; TICAM1; TLR2; TLR4; TLR7; TLR8; TLR9; TOMM70A; TRADD; TRAF3IP3; TRIM56; TRIM65; ZBTB20; ZC3HAV1
**NK inhibitory ligand gene set**	HLA-A; HLA-B; HLA-C; HLA-E.

Table S4. Sequence of genetic components of membrane-bound IL-21 engineered for aAPC.

**Components**	**Gene sequence**
**IL-21**	ATGAGATCCAGTCCTGGCAACATGGAGAGGATTGTCATCTGTCTGATGGT<br>CATCTTCTTGGGGACACTGGTCCACAAATCAAGCTCCCAAGGTCAAGATC<br>GCCACATGATTAGAATGCGTCAACTTATAGATATTGTTGATCAGCTGAAA<br>AATTATGTGAATGACTTGGTCCCTGAATTTCTGCCAGCTCCAGAAGATGT<br>AGAGACAAACTGTGAGTGGTCAGCTTTTTCCTGTTTTCAGAAGGCCCAAC<br>TAAAGTCAGCAAATACAGGAAACAATGAAAGGATAATCAATGTATCAAT<br>TAAAAAGCTGAAGAGGAAACCACCTTCCACAAATGCAGGGAGAAGACA<br>GAAACACAGACTAACATGCCCTTCATGTGATTCTTATGAGAAAAAACCAC<br>CCAAAGAATTCCTAGAAAGATTCAAATCACTTCTCCAAAAGATGATTCAT<br>CAGCATCTGTCCTCTAGAACACACGGAAGTGAAGATTCC
**IgG1**	GACAAAACTCACACATGCCCACCGTGCCCAGCACCTGAACTCCTGGGGG<br>GACCGTCAGTCTTCCTCTTCCCCCCAAAACCCAAGGACACCCTCATGATC<br>TCCCGGACCCCTGAGGTCACATGCGTGGTGGTGGACGTGAGCCACGAAG<br>ACCCTGAGGTCAAGTTCAACTGGTACGTGGACGGCGTGGAGGTGCATAA<br>TGCCAAGACAAAGCCGCGGGAGGAGCAGTACAACAGCACGTACCGTGTG<br>GTCAGCGTCCTCACCGTCCTGCACCAGGACTGGCTGAATGGCAAGGAGT<br>ACAAGTGCAAGGTCTCCAACAAAGCCCTCCCAGCCCCCATCGAGAAAAC<br>CATCTCCAAAGCCAAAGGGCAGCCCCGAGAACCACAGGTGTACACCCTG<br>CCCCCATCCCGGGAGGAGATGACCAAGAACCAGGTCAGCCTGACCTGCC<br>TGGTCAAAGGCTTCTATCCCAGCGACATCGCCGTGGAGTGGGAGAGCAA<br>TGGGCAGCCGGAGAACAACTACAAGACCACGCCTCCCGTGCTGGACTCC<br>GACGGCTCCTTCTTCCTCTACAGCAAGCTCACCGTGGACAAGAGCAGGTG<br>GCAGCAGGGGAACGTCTTCTCATGCTCCGTGATGCACGAGGCTCTGCACA<br>ACCACTACACGCAGAAGAGCCTCTCCCTGTCTCCGGGTAAA

**CD8**	ACCACGACGCCAGCGCCGCGACCACCAACACCGGCGCCCACCATCGCGT<br>CGCAGCCCCTGTCCCTGCGCCCAGAGGCGTGCCGGCCAGCGGCGGGGGG<br>CGCAGTGCACACGAGGGGGCTGGACTTCGCCTGTGATATCTACATCTGGG<br>CGCCCTTGGCCGGGACTTGTGGGGTCCTTCTCCTGTCACTGGTTATCACCC<br>TTTACTGC
**4-1BB**	AAACGGGGCAGAAAGAAACTCCTGTATATATTCAAACAACCATTTATGA<br>GACCAGTACAAACTACTCAAGAGGAAGATGGCTGTAGCTGCCGATTTCC<br>AGAAGAAGAAGAAGGAGGATGTGAA
**CD3ζ**	CTGAGAGTGAAGTTCAGCAGGAGCGCAGACGCCCCCGCGTACCAGCAGGGCCAGAACCA<br>GCTCTATAACGAGCTCAATCTAGGACGAAGAGAGGAGTACGATGTTTTGGACAAGAGACG<br>TGGCCGGGACCCTGAGATGGGGGGAAAGCCGAGAAGGAAGAACCCTCAGGAAGGCCTGT<br>ACAATGAACTGCAGAAAGATAAGATGGCGGAGGCCTACAGTGAGATTGGGATGAAAGGC<br>GAGCGCCGGAGGGGCAAGGGGCACGATGGCCTTTACCAGGGTCTCAGTACAGCCACCAA<br>GGACACCTACGACGCCCTTCACATGCAGGCCCTGCCCCCTCGC

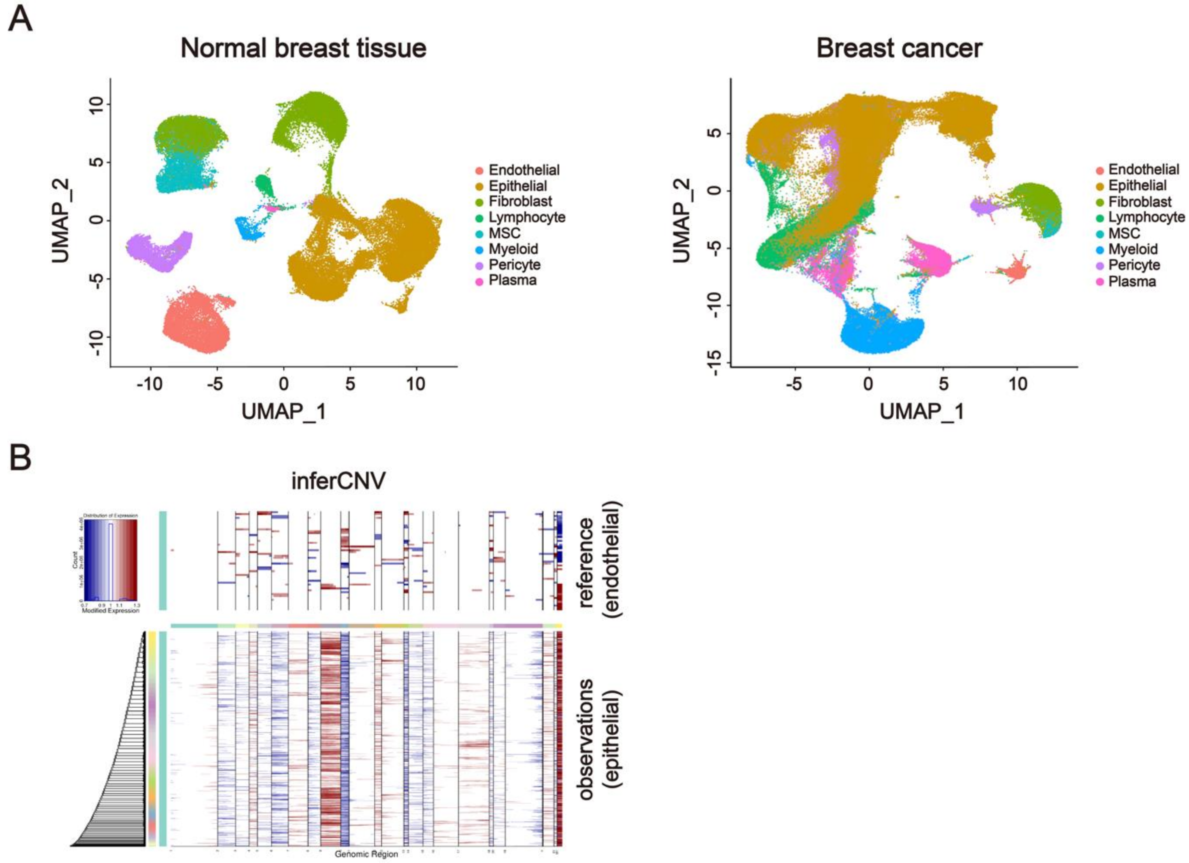

Figure S1. scRNA-seq analysis of human normal breast tissues and breast cancer. (A) UMAP visualization of 63, 970 cells from human normal breast tissues and 245, 073 cell from human breast cancer analyzed via scRNA-seq. Clusters are annotated for their cell types using canonical markers. (B) InferCNV (see Methods) results indicating that cancerous epithelial cells (reflected by CNV-high epithelial cells) are distinguished from normal epithelial cells. Endothelial cells and epithelial cells serve as a reference and observations, respectively.

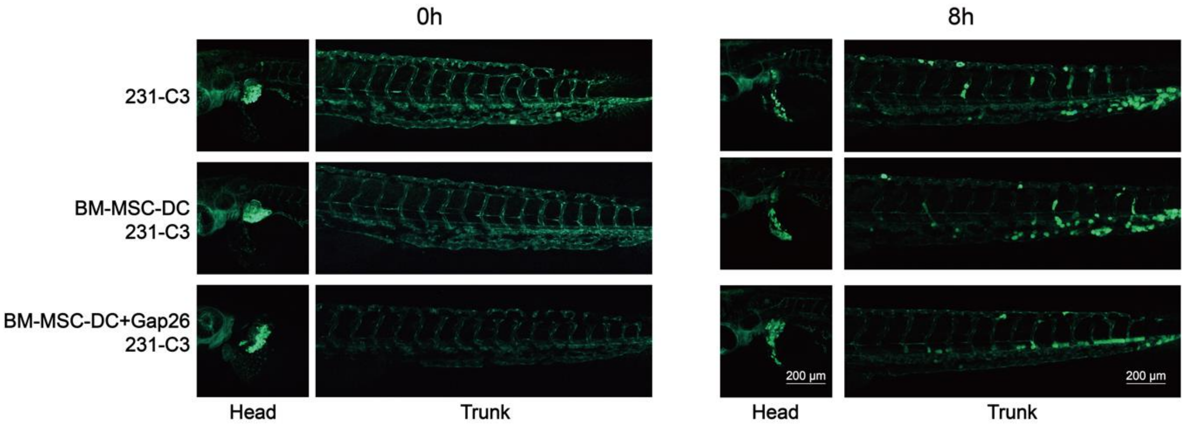

Figure S2. Viability of 231-C3 cancer cells injected into zebrafish with or without prior BM-MSC-DC and Gap26 treatment. Representative microscopy images of zebrafish are shown at 0 and 8 h post-injection. The vasculature of the animals and live 231-C3 cells were stained green and apoptotic 231-C3 cells blue which were rare in all the groups. Scale bar: 200 μm.

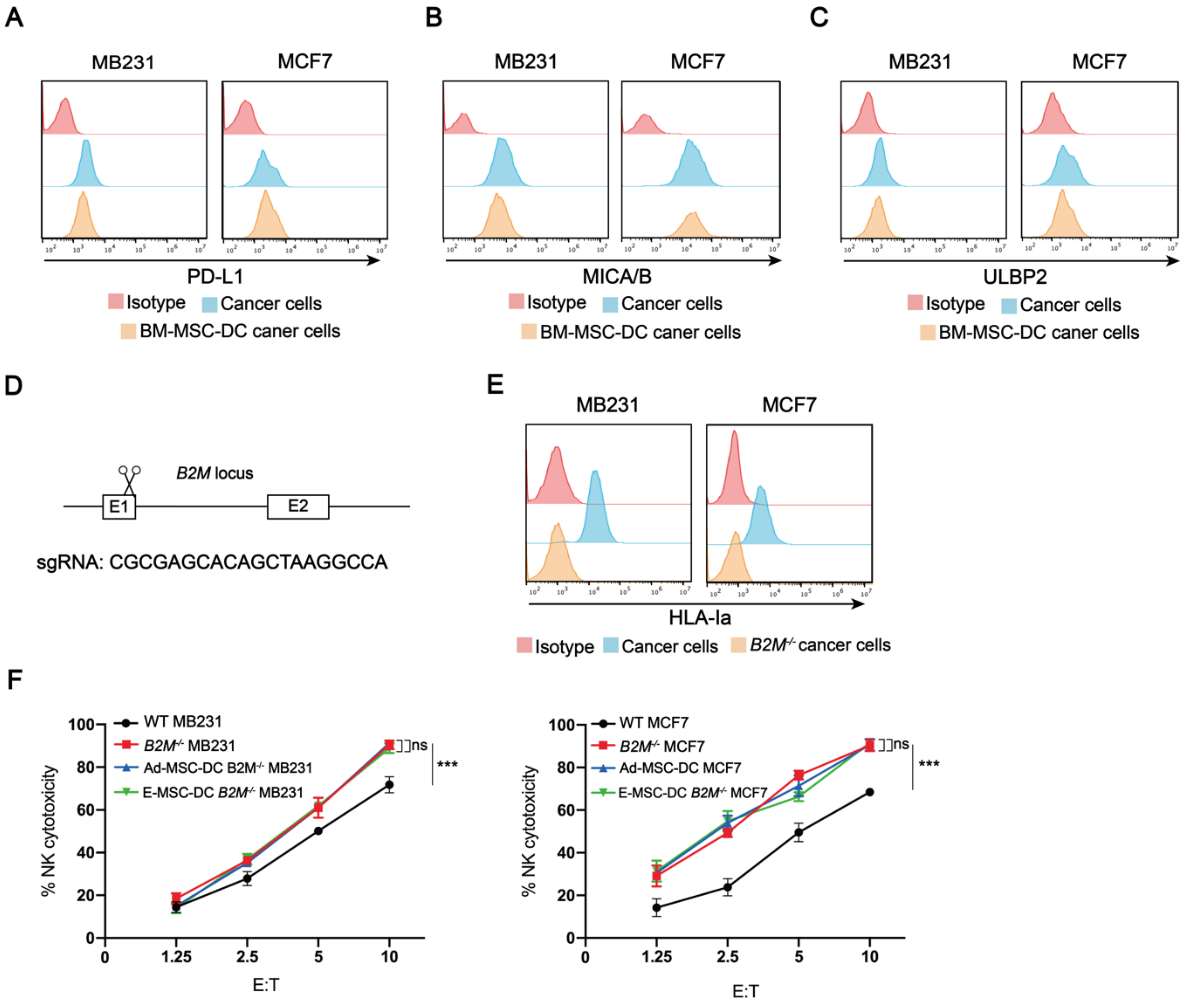

Figure S3. *B2M* knockout (KO) abolishes MSC-DC-induced NK resistance in cancer cells. (A-C) Flow cytometry for PD-L1 (**A**) , MICA/B (**B**), and ULBP2 (**C**) expression on the surface of MB231 and MCF7 cancer cells following DC with BM-MSCs for 3 days. **(D)** Schematic for *B2M* KO to generate *B2M*$^{-/-}$ MB231 and MCF7 cells. **(E)** Flow cytometry analysis for HLA-Ia expression on the surface of WT and *B2M*$^{-/-}$ cancer cells. **(F)** NK cytotoxicity assay on WT and *B2M*$^{-/-}$ cancer cells following DC with Ad- and E-MSCs. N = 3 per two-way ANOVA with multiple comparisons. Data are presented as means ± SD.

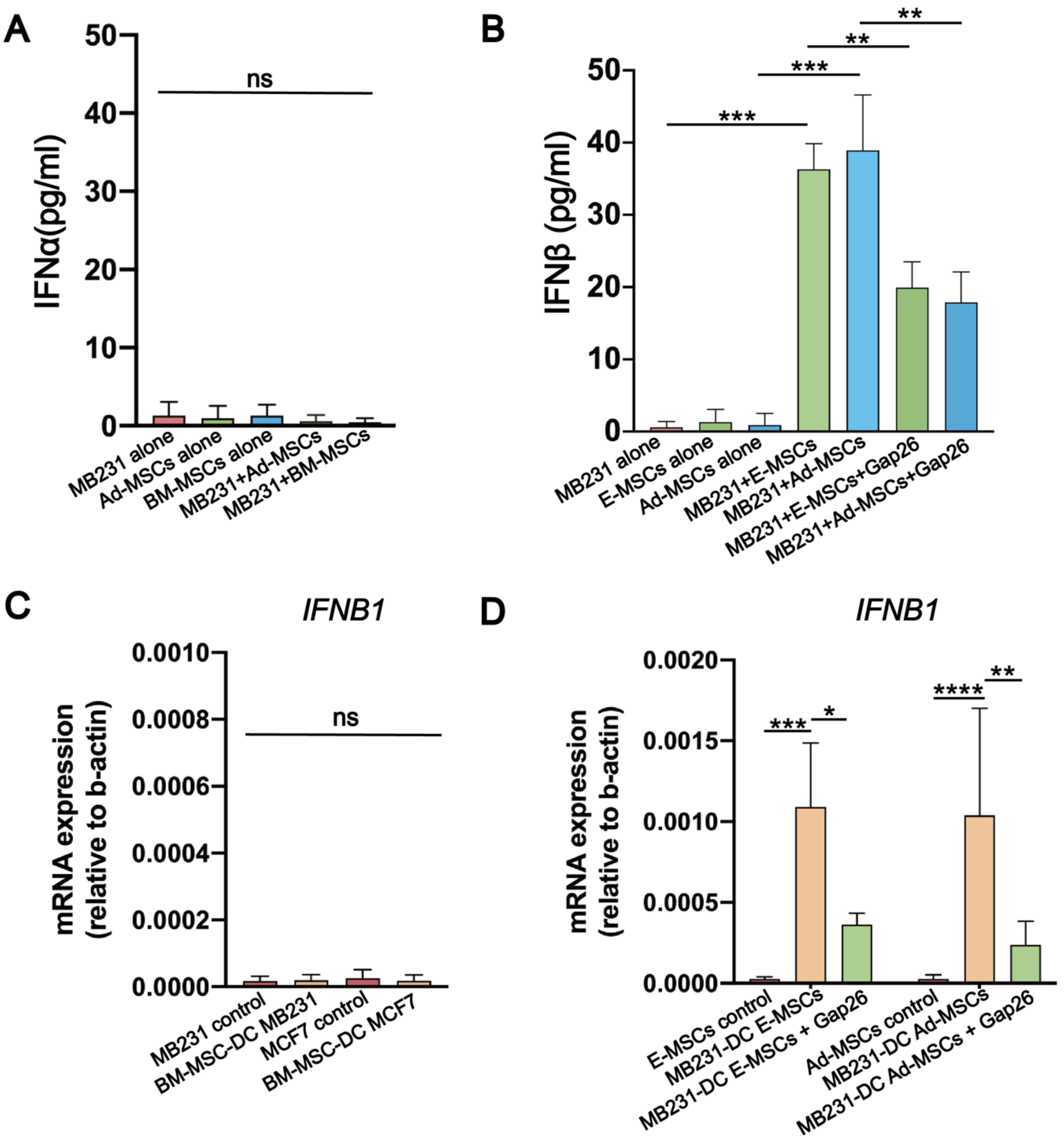

Figure S4. IFN production in MSCs and cancer cells following their DC. (A & B) ELISA for IFNα (**A**) and -β (**B**) in cancer cell culture alone, BM-MSC culture alone, DC of MSCs and cancer cells +/- Gap26. N = 3. $^{**}P < 0.01$, $^{***}P < 0.001$ per two-tailed unpaired *t* test. (**C & D**) qPCR analysis for *IFNB1* expression in cancer cells (**C**) and MSCs (**D**) each alone or following MSC-DC +/- Gap26. N = 3, $^{*}P < 0.05$, $^{**}P < 0.01$, and $^{*}P < 0.05$ per two-tailed unpaired *t* test.

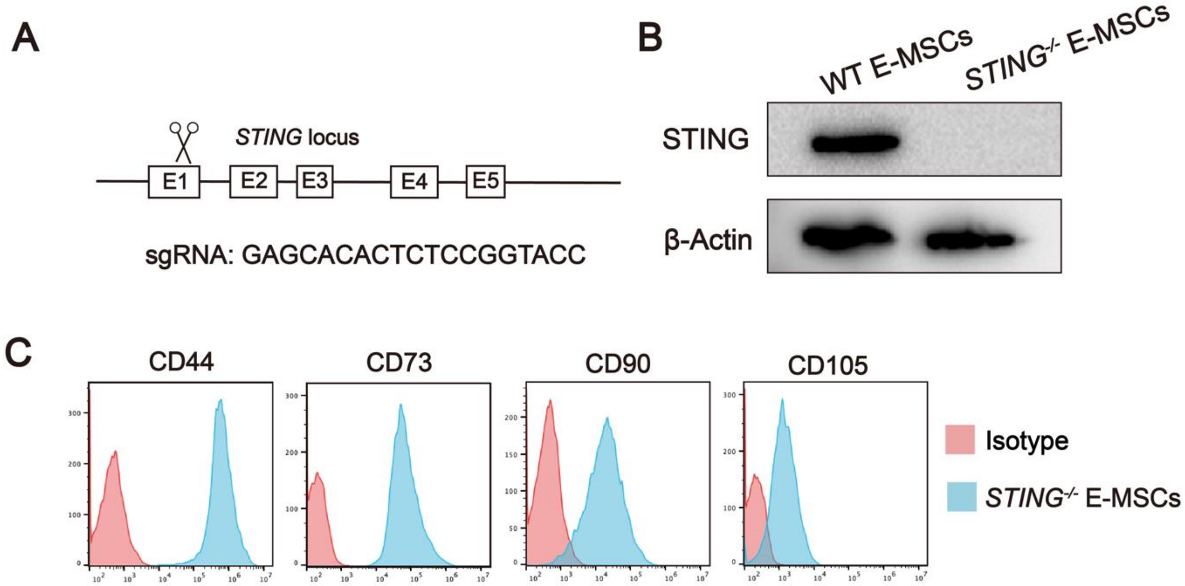

Figure S5. Generation of *STING* KO E-MSCs. (A) Schematic for generation of *STING*$^{-/-}$ hESCs. **(B)** Western blotting showing *STING*$^{-/-}$ in E-MSCs. **(C)** Flow cytometry analysis for CD44, CD73, CD90, and CD105 expression on the surface of *STING*$^{-/-}$ E-MSCs.

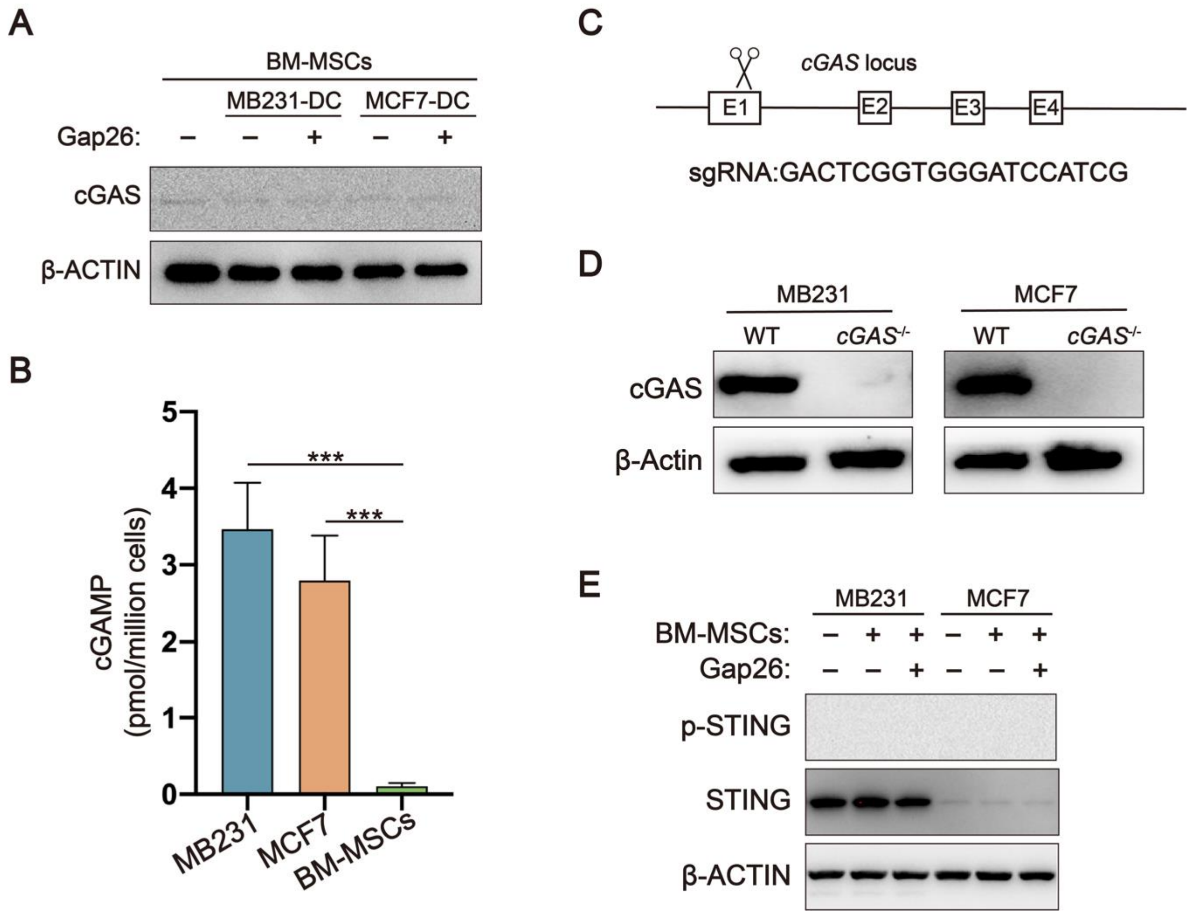

Figure S6. Detection of cGAMP and generation of *cGAS* KO cancer cells. (A) Western bloting for cGAS expression in BM-MSCs following DC with or without cancer cells and Gap26 for 3 days. **(B)** ELISA for cGAMP in the lysates of cancer cells or BM-MSCs alone. **(C)** Schematic for generation of *cGAS*$^{-/-}$ cancer cells. **(D)** Western blotting showing *cGAS* KO in cancer cells. (**E**) Western bloting for STING and phosphorylated STING (p-STING) in cancer cells following DC with or without MSCs and Gap26 for 3 days.

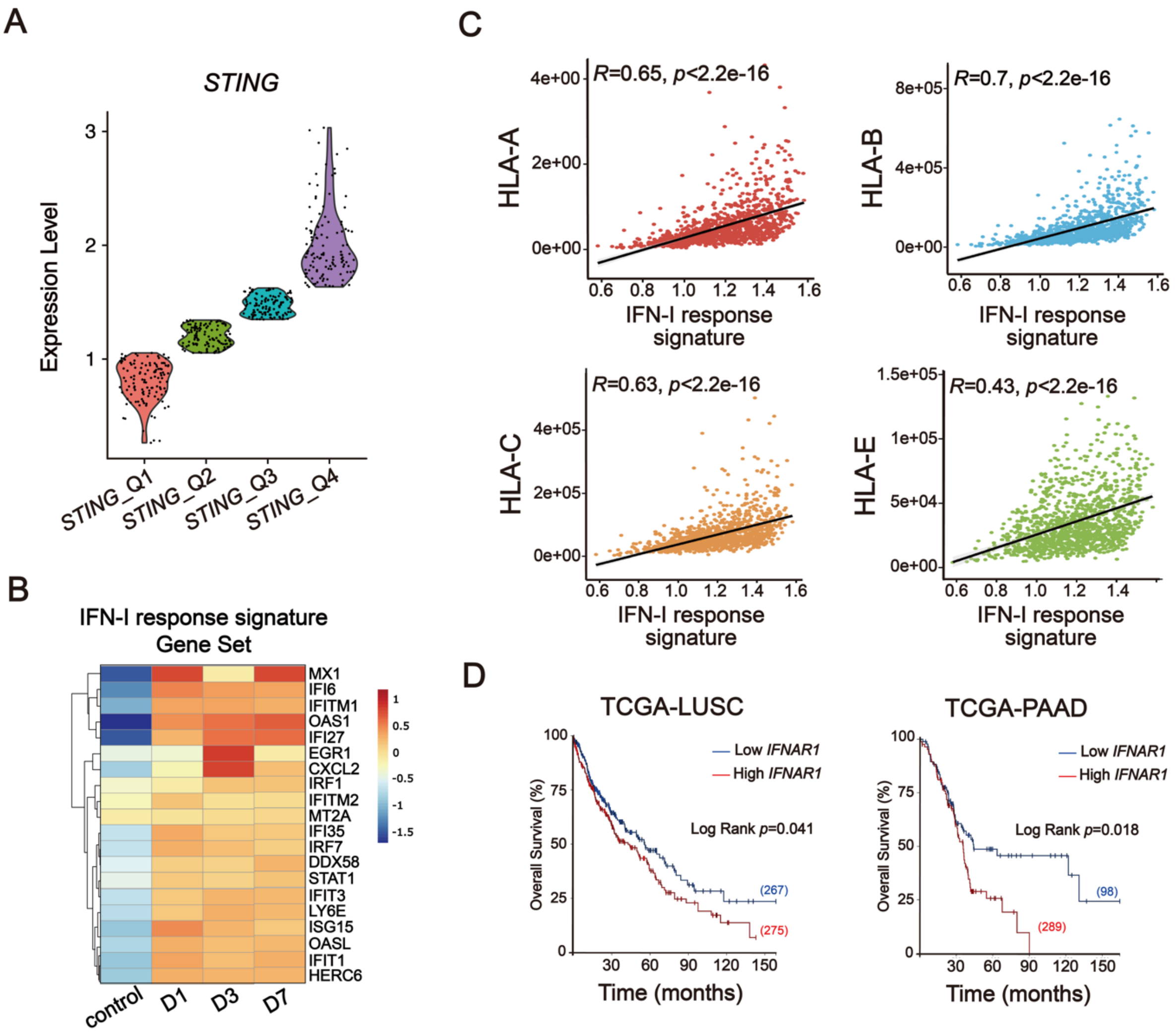

Figure S7. IFN-I signaling in human cancer samples and its correlation with patient survival.

(**A**) Violin plots showing *STING* expression level in four groups (Q1-4) of breast cancer MSCs based on scRNA-seq. (**B**) Heatmap for the relative expression level of 20 hallmark genes of the IFN-I response signature based on RNA-seq of MB231 cells following DC with MSC for 1, 3, and 7 days. (**C**) Transcriptional correlation between the expression level of HLA-I genes and IFN-I response signature genes in human breast cancer. Each dot represents a breast cancer cell from clinical samples. (**D**) Kaplan-Meier plots for the overall

survival of patients with lung squamous cell carcinoma (LUSC) and pancreatic adenocarcinoma (PAAD) based on the expression level of *IFNAR1* in the cancer cells.
